# Use and reporting of patient-reported outcomes in randomized controlled trials in non-Hodgkin lymphoma: a scoping review

**DOI:** 10.1186/s41687-026-00999-1

**Published:** 2026-01-28

**Authors:** Julia Schroer, Paul Jan Bröckelmann, Sam Salek, Tatyana Ionova, Edward Laane, Esther Natalie Oliva, Ina Monsef, Nicole Skoetz, Mario Csenar

**Affiliations:** 1 Institute of Public Health, Faculty of Medicine and University Hospital Cologne, University of Cologne, Cologne, Germany; 2 Department I of Internal Medicine, Center for Integrated Oncology Aachen Bonn Cologne Duesseldorf (CIO ABCD), Faculty of Medicine and University Hospital Cologne, University of Cologne, Cologne, Germany; 3 School of Health, Medicine and Life Sciences, University of Hertfordshire, Hatfield, UK; 4 Quality of Life Monitoring Unit, Saint Petersburg State University Hospital, Saint Petersburg, Russian Federation; 5 Hematology-Oncology Clinic, University of Tartu, Tartu, Estonia; 6 Hematology Department, London North West University Healthcare NHS Trust, London, UK

**Keywords:** Non-Hodgkin lymphoma, Patient-reported outcomes, Patient-reported experience, PRO instrument, Quality of life, Scoping review, Sankey diagram

## Abstract

**Background:**

Non-Hodgkin lymphoma (NHL) constitutes a biologically and clinically heterogeneous group of lymphoid malignancies, with varying prognoses and treatment aims between indolent and aggressive subtypes. While survival outcomes remain key efficacy measures, they insufficiently capture the impact of disease and treatment on daily physical and psychosocial functioning, as well as quality of life (QoL).

**Objectives:**

To characterize the use and reporting of patient-reported outcomes (PROs) in adult NHL randomized controlled trials (RCTs), including the trajectory of PRO dissemination throughout the trial reporting process.

**Methods:**

MEDLINE, CENTRAL, ClinicalTrials.gov, and WHO ICTRP were systematically searched from 1 January 2017 to 19 January 2023 for NHL RCTs irrespective of subtype, with databases being re-searched until 4 February 2025. Cross-sectional and longitudinal descriptive analyses were conducted to investigate PRO use and reporting practices.

**Findings:**

549 completed and ongoing RCTs met the eligibility criteria, of which 176 trials (32.1%) included a reference to PROs, and 27 (4.9%) used a PRO as a primary outcome. Overall, 95 PRO measures were identified across trials, with EORTC QLQ-C30, FACT-Lymphoma, and EQ-5D being referenced most frequently. Among trials with published results that pre-specified PROs, 25.0% entirely and 10.5% partly omitted PRO findings from their main full-texts, without addressing this shortcoming in subsequent publications.

**Conclusion:**

PROs are underused and underreported in RCTs in NHL. Standardized PRO evaluation methodology and guidance for the selection of fit-for-purpose PRO measures are needed, along with clear positioning of PROs alongside survival outcomes in clinical trial design and interpretation.

**Supplementary Information:**

The online version contains supplementary material available at 10.1186/s41687-026-00999-1.

## Introduction

Non-Hodgkin lymphoma (NHL) comprises malignant neoplasms that arise from lymphoid tissues, typically originating from the lymph nodes, but also extranodal lymphoid sites [1]. In 2022, NHL ranked the cancer types with the tenth highest incidence globally, with 553,010 incident cases (2.8% of all sites), and was the 11th leading cause of cancer-related mortality, accounting for 250,475 cancer deaths (2.6% of all sites) [2]. NHL is a highly heterogeneous group of lymphoid malignancies, characterized by notably distinct prognoses and therapeutic objectives across the indolent and aggressive subtypes [1]. While in many cases durable responses can be achieved, and the number of survivors is increasing [3], treatments are frequently associated with acute and long-term side effects [4]. Historically, the advent of the anti-CD20 monoclonal antibody rituximab transformed the treatment of B-cell lymphoma, markedly improving outcomes in an otherwise largely incurable disease [5, 6]. With the advancement of NHL therapies, increasingly moving away from conventional chemo- and/or radiotherapy towards targeted approaches [7], it is crucial to focus on the challenges affected patients may face during treatment, at early and long-term follow-up regarding physical, emotional, and social functioning. Currently, treatment goals for NHL are to improve survival while minimizing sequelae and enhancing quality of life (QoL) and other patient-reported outcomes (PROs). Although survival outcomes, such as progression-free survival and overall survival, remain the gold standard for evaluating the efficacy of treatments, they do not fully capture the impact of disease and treatment on daily physical and psychosocial functioning and QoL of a patient from their perspective.

PRO measures (PROMs) are standardized questionnaires and scales that collect information on health outcomes directly from patients [8]. This scoping review primarily focused on PROMs, which capture the patients’ perspectives on their health status, symptoms, and functional abilities. However, to provide a more comprehensive overview of patient-centered assessment instruments, patient-reported experience measures (PREMs), which reflect the patients’ experiences and satisfaction with healthcare delivery, were considered as well. PREMs were included and categorized alongside PROMs, acknowledging the conceptual distinction between the two, but recognizing their complementary roles in evaluating patient-reported health-related outcomes and healthcare quality [9].

The assessment of PROs in clinical trials has great potential to advance patient-centered care in hematology. Nonetheless, for this potential to be realized, PROs must be thoughtfully selected, rigorously measured, and transparently reported in both clinical research and practice [10, 11]. We conducted an extensive scoping review to characterize the use and reporting of PROs in randomized controlled trials (RCTs) in adult NHL, aiming to address the lack of systematic evaluations spanning the full range of NHL subtypes. Our objectives were to examine which and how many PROMs are used, how PROs are positioned within the outcome hierarchy, and how consistently and transparently they are reported, including whether PRO results are published concurrently with other trial outcomes or follow a delayed trajectory in the dissemination process.

Together with a systematic review of the validation of PRO instruments in NHL, the findings of this scoping review are intended to provide a solid foundation for the development of targeted and practice-oriented recommendations for the use and reporting of PROs in adult NHL clinical trials. This scoping review forms part of a larger guidelines development project implemented by the European Hematology Association (EHA) [12], aimed at developing guidance for the use and reporting of PROs in RCTs across various hematologic malignancies.

## Methods

This scoping review was conducted according to the updated scoping review methodology guidance of the Joanna Briggs Institute [13] and followed the Preferred Reporting Items for Systematic Reviews and Meta-Analyses extension for scoping reviews (PRISMA-ScR) (Appendix 1) [14]. References were managed in EndNote21^®^, adhering to the guideline for reference management in EndNote in the frame of systematic reviews published by Bramer and colleagues [15]. This scoping review is jointly registered with a forthcoming systematic review of validation studies of PROMs used in NHL RCTs in PROSPERO (CRD42023398574).

### Search strategy and selection criteria

An experienced information specialist (IM) developed and applied a systematic search strategy for the identification of RCTs investigating NHL. Systematic literature searches were performed in the databases MEDLINE (via Ovid) and the Cochrane Central Register of Controlled Trials (CENTRAL), as well as in the clinical trial registries ClinicalTrials.gov and the WHO International Clinical Trials Registry Platform (ICTRP). We conducted an initial comprehensive literature search spanning the period from 1 January 2017 to 19 January 2023. Following methodological guidance outlined in the Cochrane Handbook [16], re-runs of the search in bibliographic databases were conducted until 4 February 2025 to identify publications of previously ongoing studies and follow-up reports of initial trial publications. This strategy was adopted as, in our experience, PRO data are commonly omitted from primary trial publications and are often published with a delay in subsequent publications. The full search strategies are provided in the supplementary material (Appendix 2).

To generate the most comprehensive overview of which PROMs are intended for use in which contexts, ongoing studies were included, with information extracted from their registry entries and protocols. Moreover, trials at any stage of the dissemination process, including those without published results, were considered informative, as one objective of this review was to examine if, when, and how PRO findings are made publicly available. The inclusion criteria were predefined as any adult patient diagnosed with NHL regardless of subtype, stage of disease, sex, ethnicity, setting, or country. We excluded trials conducted in mixed hematologic or hemato-oncologic populations. No restrictions were imposed on interventions or trial outcomes. However, reports of trials that exclusively evaluated biochemical and prognostic factors were excluded as their emphasis on molecular endpoints did not align with the review’s clinical focus. Any type of patient-level RCT was eligible, including RCTs with a post-randomization crossover proportion or open-label extension, if these were a fixed part of the RCT which met our eligibility criteria. We considered various types of publications, covering conference abstracts and registry entries, while excluding clinical study reports. No language restrictions were applied.

### Data collection

**Fig. 1:**
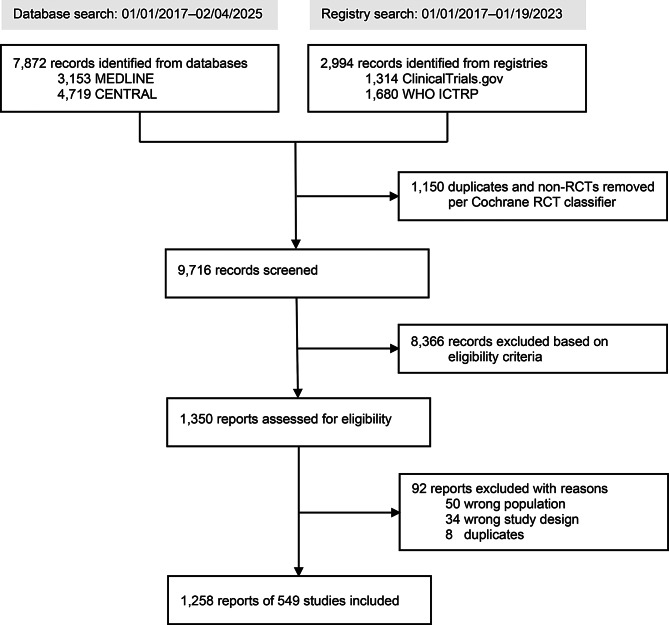
PRISMA flow diagram. The flow chart combines the initial search in bibliographic databases and clinical trial registries (01/01/2017 to 01/19/2023) and re-runs of the database search (until 02/04/2025)

Two researchers (JS, MC) independently screened the records identified through the systematic searches for RCTs and resolved any disagreements by discussion. The process of trial and record selection, including search counts and reasons for exclusions, is outlined in a PRISMA flow diagram (Fig. 1).

Information was retrieved from registry entries, trial protocols, and trial publications, and assembled for each trial. During data charting [17], two researchers (JS, MC) gathered and verified data using a pre-defined charting sheet guided by the main outcomes of the review and informed by the checklists for collecting data provided in the Cochrane Handbook [18]. Captured items included general trial information (trial name, registration details, source, sponsor, completion and publication status), data about the study design (phase, blinding, center count, follow-up), patient characteristics (NHL subtype, eligible age span, disease setting (newly diagnosed, relapsed or refractory, survivor)), experimental and comparator interventions, along with data about the primary as well as relevant secondary and exploratory outcomes. Regarding subtypes of NHL, we differentiated B-cell lymphomas from T-cell and natural killer (NK)-cell lymphomas. B-cell lymphomas were further divided into aggressive and indolent B-cell lymphomas, or mantle cell lymphoma (MCL). MCL exemplifies a special case, as there is no uniform definition of indolent and non-indolent subtypes in MCL, and the treatment approach and intensity can change during the course of the disease [19]. While lymphoid malignancies more broadly may include plasma cell neoplasms like multiple myeloma or chronic lymphocytic leukemia as non-Hodgkin subtypes, these entities were not evaluated as part of this review, which focused on nodal, non-leukemic variants. Key trial characteristics were assessed to contextualize the use and reporting of PROs. Intending to explore the frequency of PROs used as outcome measures (primary, secondary, or exploratory), we investigated whether the measurement of PROs was prospectively planned, which instruments were planned to be or were used, and if there were any deviations concerning the reporting PROs in related trial publications. No missing data was deemed relevant enough to request further information or undisclosed data from the study authors.

### Evidence synthesis

We performed a narrative synthesis of the findings across trials. Prior to synthesis, data were categorized according to pre-defined variables to facilitate analysis and structured comparison. Qualitative variables were summarized by absolute (n) and relative (%) level frequency, and visualized as bar charts, a Sankey diagram, or a heatmap. In multiple studies, mixed NHL populations, such as indolent B- and T-cell lymphomas, were investigated. To clearly identify which instruments were used in different subtypes of NHL, a threshold of 80% of the study population was used to explicitly classify the population as one of the NHL subtypes. Mixed study populations without clear majority of an NHL subtype remained classified as mixed. We categorized the study sponsor, defined as the organization or person who initiates the study and has authority and control over it [20], as either an industry sponsor (a for-profit organization) or a non-industry sponsor (a non-profit, academic, and/or public institution) as declared in the clinical trial registry entry. Importantly, categorization as a non-industry sponsor did not preclude the involvement of a for-profit collaborator providing financial or in-kind support such as study drug supply. For the assessment of PRO reporting consistency, we assessed the trial completion status and concordance of registry entries and, if accessible, protocols with related full-text publications. In the absence of published results, we proceeded with PROMs intended for use.

Our descriptive analysis focused on three distinct aspects of PROs and PROMs in RCTs. First, the use of PROMs was investigated by exploring the frequency of distinct PROMs and their scope of utilization (generic, condition-specific, symptom- or side-effect-specific, patient-reported experience and satisfaction). Second, the frequency of use and reporting of PROs was evaluated, including their positioning in the hierarchy of trial outcomes, context of use, and consistency of reporting. Third, PRO reporting was evaluated over a two-year period to assess whether newly published results had emerged for previously identified RCTs, particularly for trials in which PROs were pre-specified but not yet reported. While the first and second aspect of analysis included the full set of identified RCTs for cross-sectional analysis, the third aspect of analysis was restricted to the subset of initially identified RCTs for which follow-up reports were sought for longitudinal analysis. Therein, we compared PRO reporting trajectories between two time points. Given the frequently encountered fragmentation of PRO reporting across separate publications, this approach was chosen to maintain coherence and feasibility. Subgroup analyses were conducted to examine the use of PROMs in different NHL subtypes.

### Role of the funding source

The funder of this review (EHA) had no role in the study design, data collection, data analysis, data interpretation, writing of the report, or in the decision to submit the paper for publication.

## Results

### Search results and included studies

Search results are illustrated in the PRISMA flow diagram (Fig. 1). The systematic database and trial registry searches yielded 10,866 records, of which 1,150 duplicates and non-RCTs, as identified by the Cochrane RCT classifier, were removed [21]. After screening the titles and abstracts of 9,716 records, 8,366 records were incompatible with the eligibility criteria and, therefore, excluded. Screening of 1,350 full-texts led to the exclusion of 92 reports. The main reasons for exclusion of full-texts were a wrong study population (e.g., mixed oncologic populations), and a wrong study design (e.g., no RCT). Finally, 1,258 reports of 549 RCTs were included in this scoping review, of which 487 trials were identified during the initial search and hence were available for longitudinal analysis. Given the large number of included studies, it was not practicable to reference the contributing trials for each individual finding in the main text. A complete list of all 549 included RCTs, along with registration numbers and sources, is provided in the supplementary material (Appendix 3).

Most of the studies were phase 3 trials (246 trials, 44.8%), followed by phase 2 trials (134 trials, 24.4%), phase 1/2 trials (31 trials, 5.6%), phase 4 trials (23 trials, 4.2%), phase 1 trials (16 trials, 2.9%), and phase 2/3 trials (13 trials, 2.4%). In 86 trials (15.7%), the trial phase was either unreported or not applicable. Most trials were open-label (405 trials, 73.8%), whereas 84 trials (15.3%) were double-blind, 24 trials (4.4%) were single-blind, and blinding details were unavailable for 36 trials (6.6%). More than three-quarters of trials (420 trials, 76.5%) were multi-center, while 117 trials (21.3%) were restricted to a single research center. For 12 trials (2.2%), the number of participating centers could not be determined. Among all NHL trials, which varied widely in study design and type of intervention, the majority were sponsored by non-industry entities (330 trials, 60.1%), including university hospitals, research foundations, and governmental agencies. A total of 187 trials (34.1%) were directly industry-sponsored, predominantly by pharmaceutical and biotechnology companies. No information about the sponsor was available for 32 trials (5.8%). Across trials with published follow-up data (192 trials, 35.0%), the median follow-up was 4.1 years.

**Fig. 2:**
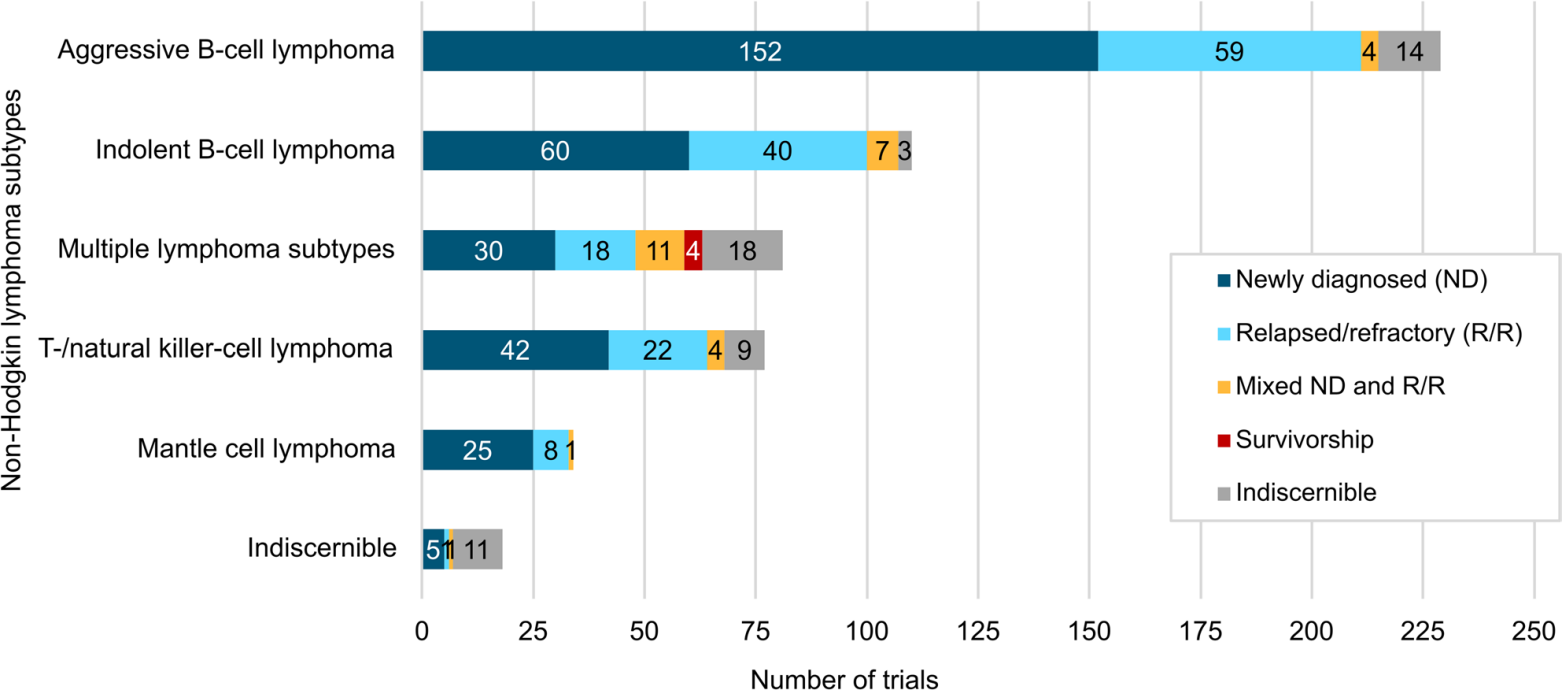
Non-Hodgkin lymphoma (NHL) subtypes and disease settings in NHL randomized controlled trials (RCT). RCTs were identified between 01/01/2017 and 02/04/2025. Newly diagnosed: ND; Relapsed/refractory: R/R

More than half of the RCTs (314 trials, 57.2%) were conducted in patients with newly diagnosed (ND) disease. Patients with relapsed or refractory (R/R) disease were participants in 148 trials (27.0%), NHL survivors in four trials (0.7%), and mixed ND-R/R populations were investigated in 28 trials (5.1%). For 55 trials (10.0%), no information about the disease setting could be retrieved. Across RCTs, the most frequently studied NHL subtype was aggressive B-cell lymphoma (229 trials, 41.7%). Diffuse large B-cell lymphoma (DLBCL), primary central nervous system lymphoma, follicular lymphoma grade 3B (largely re-classified as follicular large B-cell lymphoma per the 5th edition of the WHO classification) [1], and primary mediastinal B-cell lymphoma were the most strongly represented aggressive B-cell lymphomas. Indolent B-cell lymphomas were investigated in 110 trials (20.0%), with follicular lymphoma grade 1 to 3 A, marginal zone lymphoma, and small lymphocytic lymphoma being the predominantly studied entities. Thirty-four trials focused on MCL (6.2%). T-/NK-cell lymphomas were explored in 77 RCTs (14.0%), with peripheral T-cell lymphoma and primary cutaneous T-cell lymphoma, including mycosis fungoides and Sézary syndrome, as well as extranodal NK/T-cell lymphoma being the most frequent. Several studies examined heterogeneous patient populations, including both aggressive and indolent B-cell lymphomas (31 trials, 5.6%), as well as those with Hodgkin or non-Hodgkin lymphoma (26 trials, 4.7%) – with NHL predominating – or combined T- and B-cell lymphomas (24 trials, 4.4%). In some instances, where NHL or malignant lymphoma was not otherwise specified, the subtype was classified as indiscernible (18 trials, 3.3%). NHL subtypes and disease settings are summarized in (Fig. 2). Notably, trials involving mixed populations or lacking a clear specification of the lymphoma subtype are also more likely to either not impose any restrictions on the disease setting in their inclusion criteria or to report these details imprecisely.

Considering the intervention regimen, approximately two-thirds of trials (351 trials, 63.9%) investigated drugs or combinations of drugs. In 107 trials (19.5%), drugs were combined with radiotherapy and/or hematopoietic stem cell transplantation, and radiotherapy alone was examined in four RCTs (0.7%). Chimeric antigen receptor (CAR) T-cell therapy was investigated in 16 trials (2.9%). Supportive interventions to enhance the effectiveness of lymphoma treatment or to mitigate the burden of undesired side effects of treatment, such as nutritional supplements or innovative nursing approaches, were studied in 48 trials (8.7%). Targeted QoL interventions, like exercise programs and patient-centered care models, were investigated in 23 RCTs (4.2%).

### PROMs incorporated in NHL RCTs

**Fig. 3:**
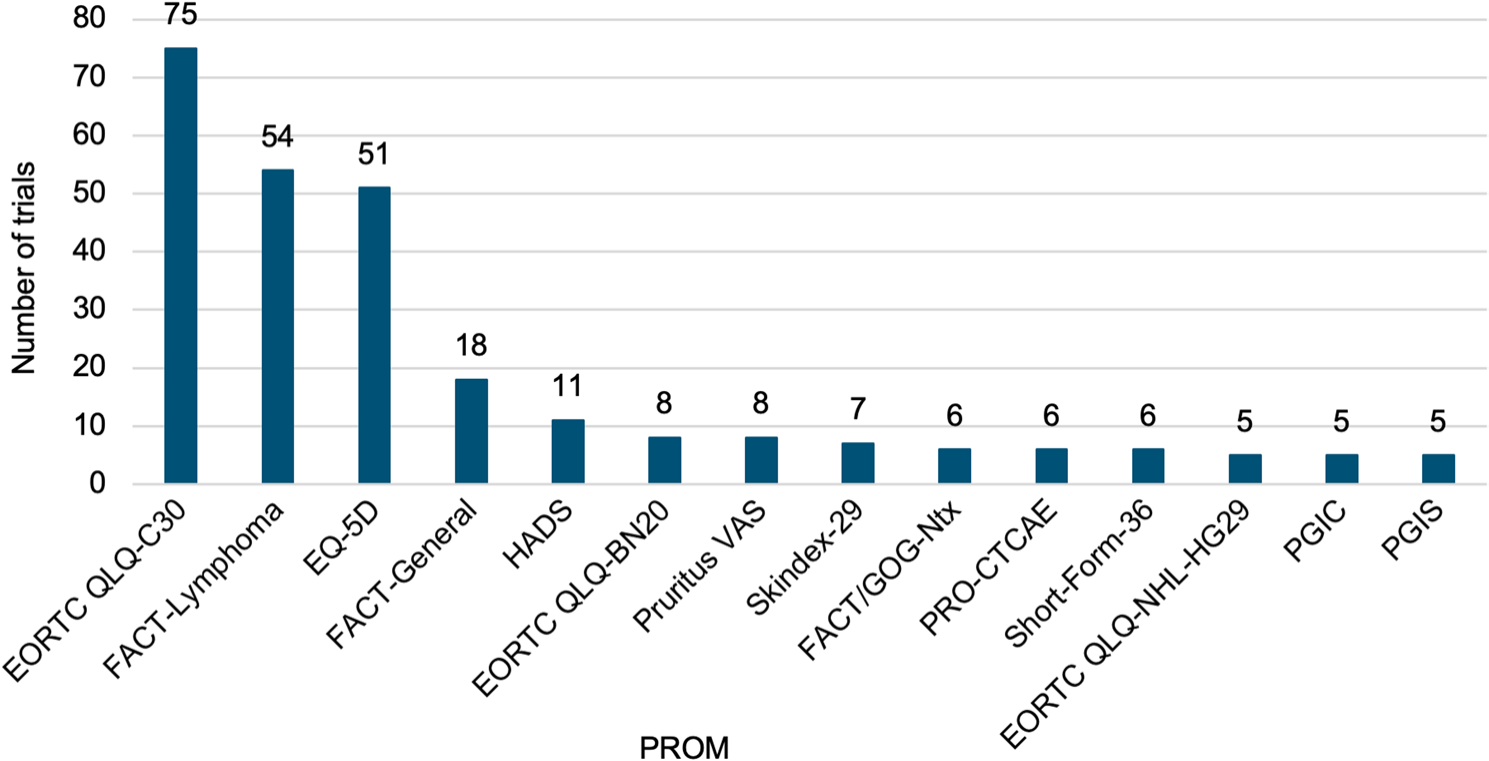
Most frequently cited patient-reported outcome measures (PROM) across NHL RCTs. RCTs were identified between 01/01/2017 and 02/04/2025. BN20: Brain Cancer module; CTCAE: Common Terminology Criteria for Adverse Events; EQ-5D: EuroQol 5 Dimensions; GOG: Gynecologic Oncology Group; HADS: Hospital Anxiety and Depression Scale; NHL-HG29: High-Grade NHL module; Ntx: Neurotoxicity; PGIC: Patient Global Impression of Change; PGIS: Patient Global Impression of Severity; VAS: Visual Analogue Scale

The cross-sectional analysis of the use of PROMs was conducted irrespective of trial completion or publication status. For ongoing trials, information on PRO utilization was incorporated from trial registries or published protocols, where available. Overall, we identified 95 PROMs that were used or planned to be used in RCTs in NHL. The PROMs were classified as generic, condition-specific, symptom- or side effect-specific, or as measures of patient experience and satisfaction, as shown in Table 1 [22]. Trials referencing each PROM are detailed in the supplementary material (Appendix 4). The most frequently mentioned PROMs are depicted in Fig. 3.

**Table 1:** Patient-reported outcome measures (PROM) identified in randomized controlled trials in non-Hodgkin lymphoma (NHL)

Scope of use	PROMs	Frequency
**Generic quality of life measures**		
	EuroQoL 5 Dimensions (EQ-5D)	51 (9%)
	General Quality of Life Inventory (GQOL-74)	1 (< 1%)
	Patient-Reported Outcomes Measurement Information System (PROMIS-29)	3 (1%)
	Short-Form-36 (SF-36)	6 (1%)
	WHO Quality of Life Scale (WHOQOL)	2 (< 1%)
**Condition-specific measures**		
	EORTC QLQ-Brain Cancer (BN20)	8 (2%)
	EORTC QLQ-Chronic Lymphocytic Leukaemia (CLL17)	1 (< 1%)
	EORTC QLQ-Core Questionnaire (C30)	75 (14%)
	EORTC QLQ-Elderly Cancer Patients (ELD14)	2 (< 1%)
	EORTC QLQ-High Dose Chemotherapy (HDC29)	1 (< 1%)
	EORTC QLQ-NHL-High Grade (HG29)	5 (1%)
	EORTC QLQ-NHL-Low Grade (LG20)	2 (< 1%)
	FACT-Anaemia (FACT-An)	1 (< 1%)
	FACT-Bone Marrow Transplant (FACT-BMT)	1 (< 1%)
	FACT-Central Nervous System (FACT-CNS)	1 (< 1%)
	FACT-General (FACT-G)	18 (3%)
	FACT-Lymphoma (FACT-Lym)	54 (10%)
	FACT/NCCN-Lymphoma Symptom Index (FLymSI-18)	3 (1%)
	FACT-Neutropenia (FACT-N)	1 (< 1%)
	Haematological Malignancy PROM (HM-PRO)	1 (< 1%)
	Kansas City Cardiomyopathy Questionnaire (KCCQ-12)	1 (< 1%)
	Quality of Life Cancer Survivor (QOL-CS)	1 (< 1%)
	WHO Quality of Life-HIV (WHOQOL-HIV)	1 (< 1%)
**Symptom- or side effect-specific**	**Symptom scales**	
**measures**		
	Edmonton Symptom Assessment Scale	1 (< 1%)
	MD Anderson Symptom Inventory (MDASI)	3 (1%)
	Memorial Symptom Assessment Scale (MSAS)	1 (< 1%)
	PRO-CTCAE (measurement system)*	6 (1%)
	Rotterdam Symptom Checklist	2 (< 1%)
	**Fatigue**	
	Brief Fatigue Inventory (BFI)	3 (1%)
	Chalder Fatigue Questionnaire (CFQ)	1 (< 1%)
	EORTC QLQ-Fatigue (FA12)	1 (< 1%)
	FACIT-Fatigue (FACIT-F)	3 (1%)
	Multidimensional Fatigue Inventory (MFI)	3 (1%)
	**Skin-related symptoms**,** including pruritus**	
	Dermatology Life Quality Index (DLQI)	4 (1%)
	ItchyQoL	1 (< 1%)
	Pruritus Quality of Life Score (PQOL)	1 (< 1%)
	Pruritus visual analogue scale (VAS)	8 (2%)
	Skindex-29	7 (1%)
	**Neurotoxicity**,** including peripheral neuropathy**	
	EORTC QLQ-Chemotherapy-Induced Peripheral Neuropathy (CIPN20)	1 (< 1%)
	FACT/GOG-Neurotoxicity (FACT/GOG-Ntx)	6 (1%)
	Therapy-Induced Neuropathy Assessment Scale (TINAS)	1 (< 1%)
	**Nausea and vomiting**	
	Functional Living Index-Emesis (FLIE)	2 (< 1%)
	**Anxiety**,** depression**,** and distress**	
	Brief Illness Perception Questionnaire (B-IPQ)	2 (< 1%)
	Cancer Worry Inventory (CWI)	1 (< 1%)
	Cancer Worry Scale (CWS)	1 (< 1%)
	Distress Thermometer (DT)	2 (< 1%)
	Generalised Anxiety Disorder Scale-7 (GAD-7)	1 (< 1%)
	Geriatric Depression Scale	1 (< 1%)
	Hospital Anxiety and Depression Scale (HADS)	11 (2%)
	Impact of Event Scale – Revised (IES-R)	2 (< 1%)
	Patient Health Questionnaire (PHQ-9)	3 (1%)
	PROMIS Short-Form Anxiety 7a	1 (< 1%)
	PROMIS Short-Form Depression 8b	1 (< 1%)
	Self-Rating Anxiety Scale (SAS)	2 (< 1%)
	Self-Rating Depression Scale (SDS)	2 (< 1%)
	Spielberger State-Trait Anxiety Scale (STAI)	2 (< 1%)
**Measures of functional status and**		
**physical activity**		
	Active Australia Survey	1 (< 1%)
	Behavioral regulation in exercise questionnaire (BREQ-3)	1 (< 1%)
	HIV Self-Management Scale	1 (< 1%)
	Godin-Shephard Leisure Time Physical Activity Questionnaire (LTPA-Q)	1 (< 1%)
	Instrumental Activities of Daily Living (IADL)	1 (< 1%)
	International Physical Activity Questionnaire (IPAQ)	2 (< 1%)
	Perceived Exercise Competence Scale (PCS)	1 (< 1%)
	Psychological need satisfaction in exercise (PNSE)	1 (< 1%)
	Self-efficacy for walking scale (SEW)	1 (< 1%)
	Short-Form Survivor Unmet Needs (SF-SUNS)	1 (< 1%)
**Measures of patient experience**	**Patient perceptions and satisfaction**	
**and satisfaction**		
	Cancer Therapy Satisfaction Questionnaire (CTSQ)	2 (< 1%)
	Consultation and Relational Empathy (CARE) measure	1 (< 1%)
	Health Change Questionnaire (HCQ)	1 (< 1%)
	Herth Hope Index (HHI)	1 (< 1%)
	Illness Perception Questionnaire-Revised (IPQ-R)	1 (< 1%)
	Information Satisfaction Questionnaire (ISQ)	1 (< 1%)
	Lima Happiness Scale	1 (< 1%)
	Patient Assessment of Chronic Illness Care (PACIC-20)	1 (< 1%)
	Patient Global Impression of Change (PGIC)	5 (1%)
	Patient Global Impression of Severity (PGIS)	5 (1%)
	Patient Preference Questionnaire (PPQ)	1 (< 1%)
	Patient Satisfaction with the Consultation (PSC)	1 (< 1%)
	Rituximab Administration Satisfaction Questionnaire (RASQ)	2 (< 1%)
	Satisfaction With Life Scale (SWLS)	1 (< 1%)
	Subjective Vitality Scale (SVS)	1 (< 1%)
	**Coping**	
	Cancer Behaviour Inventory (CBI)	1 (< 1%)
	Health Education Impact Questionnaire (heiQ)	1 (< 1%)
	Illness Coping Strategies Scale (ICS)	1 (< 1%)
	Mental Adjustment to Cancer (MAC)	3 (1%)
	Strategies Used by People to Promote Health (SUPPH)	1 (< 1%)
	**Nutrition**	
	Norwegian Digital Food-Frequency Questionnaire (DIGIKOST-FFQ)	1 (< 1%)
	Short Nutritional Assessment Questionnaire (SNAQ)	1 (< 1%)
	**Sleep**	
	Petersburg Sleep Questionnaire (PSQI)	1 (< 1%)
	Sleep Disturbance visual analogue scale (VAS)	1 (< 1%)
	Verran and Snyder-Halpern Sleep Scale (VSH)	1 (< 1%)
	**Work and finances**	
	Employment and Health Services Questionnaire (EHSQ)	1 (< 1%)
	FACIT-Cost	1 (< 1%)
	Trøndelag Health Study (HUNT) Work Status	1 (< 1%)
	Work Ability Index (WAI)	2 (< 1%)

* PRO-CTCAE is a patient-reported outcome measurement system that can provide a flexible fit-for-purpose approach to assess relevant symptomatic adverse events in patients on cancer clinical trials

Two QoL measurement systems were frequently used in NHL RCTs: the modular system of the European Organization for Research and Treatment of Cancer (EORTC) and the system of the Functional Assessment of Chronic Illness Therapy (FACIT) group. Both systems are based on a core questionnaire for the assessment of health-related QoL of cancer patients in general, i.e., the 30-item EORTC QLQ-C30 [23] and the 27-item Functional Assessment of Cancer Therapy (FACT)-General (FACT-G) questionnaire [24]. The EORTC QLQ-C30 was complemented by various modules capturing the condition- and symptom-specific impact of diseases on QoL, as well as the side effect-specific impact of treatments. While EORTC QLQ-C30 was referenced in 75 RCTs, the four most frequently named EORTC QLQ-modules were the Brain Cancer module (QLQ-BN20, 8 trials), the NHL-specific NHL-High Grade (QLQ-NHL-HG29, 5 trials) and NHL-Low Grade modules (QLQ-NHL-LG20, 2 trials), as well as the Elderly Cancer Patients module (QLQ-ELD14, 2 trials). FACT-G was occasionally used alone (18 trials), and frequently complemented by the lymphoma-specific subscale FACT-Lymphoma (FACT-Lym, 54 trials), neurotoxicity subscale (FACT/GOG-Ntx, 6 trials), and fatigue subscale (FACIT-F, 3 trials).

For all NHL subtypes, except for T-/NK-cell lymphoma, the EORTC QLQ-C30 questionnaire was among the three most cited PROMs. In aggressive and indolent B-cell lymphoma as well as MCL, the two other most common PROMs were the FACT-Lym and EQ-5D, while in trials in patients with indiscernible NHL subtype, FACT-Lym and the Hospital Anxiety and Depression Scale (HADS) were leading. In T-/NK-cell lymphoma, the pruritus visual analogue scale, Skindex-29, EQ-5D, Dermatology Life Quality Index (DLQI), and FACT-G emerged most often.

### Use and reporting frequency of PROs in NHL RCTs

Overall, 176 of 549 completed or ongoing RCTs in NHL (32.1%) considered PRO(s) as trial outcomes, whereas 373 trials (67.9%) did not indicate any intention to collect and report PROs. Of 76 trials that pre-specified PROs and for which a full-text was available, 19 (25.0%) omitted PRO reporting entirely, while eight (10.5%) only partially reported PROs, omitting single PROMs. Forty-nine RCTs (64.5%) consistently reported PROs, listing results for all PROMs intended for use. Notably, the depth and detail of use and reporting varied across trials, and none of the trials reporting PRO results cited the Consolidated Standards of Reporting Trials (CONSORT) PRO extension. In the absence of a full-text publication, reporting could not be assessed for 93 RCTs that planned to collect PROs according to their protocol or registry entry. For seven trials lacking a full-text publication, PROs were reported in an abstract. For 214 trials (39.0%), no information was available regarding planned PRO collection from their protocol or registry entry, nor was any full-text available. Utilization of PROs was neither planned nor reported in the case of 159 trials (29.0%).

**Fig. 4:**
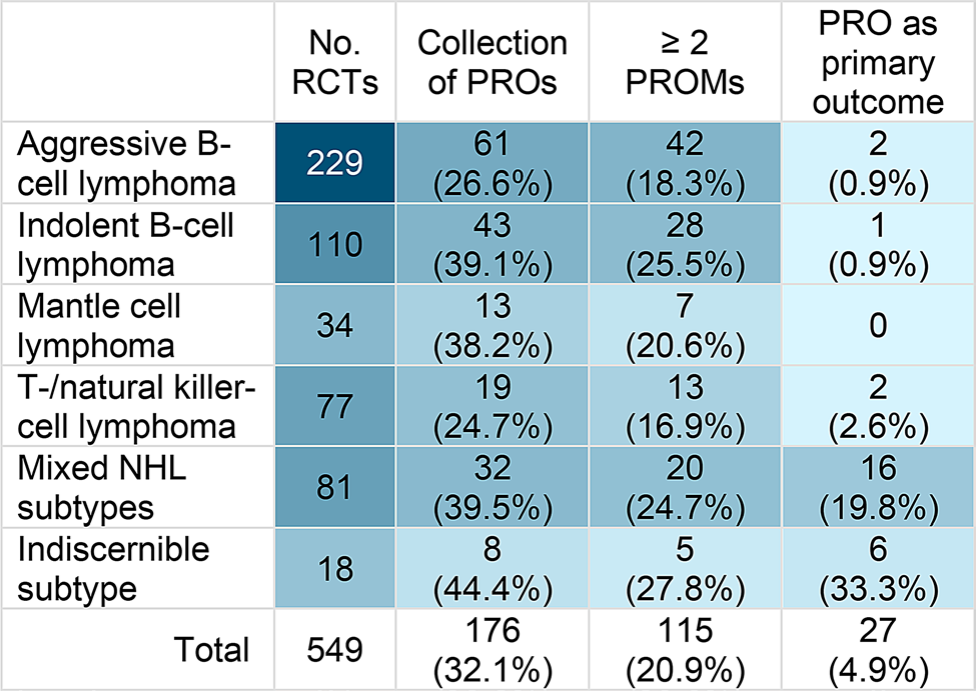
Use of patient-reported outcomes (PRO) in RCTs across NHL subtypes irrespective of publication status. Relative values (%) refer to the total number of RCTs within each NHL subgroup as the respective denominator

In 27 RCTs (4.9%), a PRO was used as a primary or co-primary outcome. Most of these trials (22 trials) investigated a QoL intervention, such as an exercise or collaborative psychological care program, aiming at improving QoL and reducing the burden of symptoms and side effects. Various instruments were employed besides broadly established generic or lymphoma-specific instruments, including fatigue-specific measures like the Multidimensional Fatigue Inventory (MFI). In three trials with a PRO as the primary outcome, supportive dietary interventions were investigated. A common characteristic of studies evaluating QoL or supportive interventions, observed in 21 cases, was the limited detail in describing the target populations or the intentional inclusion of heterogeneous populations. Only two interventional drug trials used a PRO as a (co-) primary outcome. The Watch and Wait trial compared watchful waiting and two rituximab strategies in asymptomatic follicular lymphoma, with QoL assessed via FACT-G and the HADS [25]. The PrefMab trial compared subcutaneous versus intravenous rituximab administration schedules alongside chemotherapy in previously untreated DLBCL or follicular lymphoma, with patient preference assessed using the Patient Preference Questionnaire (PPQ) as the primary outcome [26]. Among the RCTs that planned or reported on PROs, 115 of 176 trials (65.3%) mentioned at least two PROMs (Fig. 4). When excluding RCTs which had a PRO as a (co-) primary outcome from the analysis, PRO reporting rates drop slightly. Among 62 trials with a full-text publication that planned to include a PRO as a secondary or exploratory outcome, 39 trials (62.9%) consistently reported PROs, five trials (8.1%) partially reported PROs, and 18 trials (29.0%) entirely omitted PRO reporting.

PROs were more commonly included as outcomes in more advanced stages of clinical testing, being intended for use in 91 phase 3 trials (51.7%) and 36 phase 2 trials (20.5%). Of 420 multi-center trials, 136 (32.4%) planned to incorporate PROs, with a similar share observed for single-center trials (36 of 117 trials, 30.8%). Notably, the majority of trials investigating a targeted QoL intervention were conducted in a single research center (14 of 23 trials, 60.9%). Many of these trials examined exercise-based interventions or collaborative care models necessitating organizational-level adaptations.

Of the 176 trials, ongoing or completed, that incorporated PROs in their study design, 93 trials (52.8%) were sponsored by a non-industry sponsor, while 71 trials (40.3%) were industry-sponsored. In 12 trials (6.8%), which predominantly examined targeted QoL interventions, sponsorship could not be identified. These numbers compare to the overall sponsor distribution among all 549 included trials, of which 60.1% were non-industry-sponsored, 34.1% industry-sponsored, and 5.8% had unspecified sponsorship, reflecting a modest shift in sponsor distribution among trials integrating PROs. Among 49 trials that consistently reported PROs in their full-text publications, 21 trials (42.8%) were industry-sponsored, 22 trials (44.9%) were non-industry sponsored, and no information about the sponsor was retrievable for six trials (12.4%). Of the 19 trials with available full-texts that entirely omitted PRO reporting, 10 (52.6%) were industry-sponsored and nine (47.4%) non-industry-sponsored. Of eight trials with full-texts that partially omitted PRO reporting, five (62.5%) were industry- and three (37.5%) were non-industry-sponsored, respectively.

Among the RCTs with registry information, PROs were more frequently incorporated into trials registered from 2020 onwards, with 64 of 177 trials (36.2%) including PROs in their design, compared to 98 of 322 trials (30.4%) registered before 2020. Notably, among the 50 unregistered studies, nine listed a PRO as the primary outcome, highlighting a lack of prospective registration despite the central role of PROs in these trials.

### Trajectory of PRO reporting by publication status

A subset of 487 trials identified during the initial search was available for longitudinal analysis, as these trials were systematically followed up to capture subsequent publications. Overall, 453 of 487 trials (93.0%) were registered in a clinical trial registry. Initially, 145 of 453 registered trials (32.0%) were completed, 204 trials (45.0%) were ongoing (i.e., recruiting, active but not recruiting, or not yet recruiting), 42 trials (9.3%) were discontinued (i.e., terminated, suspended, or withdrawn), and 62 trials (13.7%) were listed with an unknown trial status. Study sponsors had not verified the trial status of all but five of the trials with an initially unknown status, suggesting that once there is prolonged inactivity in verification of the trial status, subsequent actualization occurs infrequently. Throughout the investigated two-year period, of the 204 RCTs that were initially ongoing, most RCTs (158 trials, 77.5%) were still ongoing and 26 trials (12.7%) were meanwhile indicated to be completed, of which four trials (15.4%) presented with a new abstract or full-text publication. Overall, during initial data charting, 236 of 387 trials (48.5%) had not been published in full-text form or otherwise. Of these, two years later, eight trials (3.4%) had a new abstract available two years later, and 11 trials (4.7%) had a new full-text publication.

**Fig. 5:**
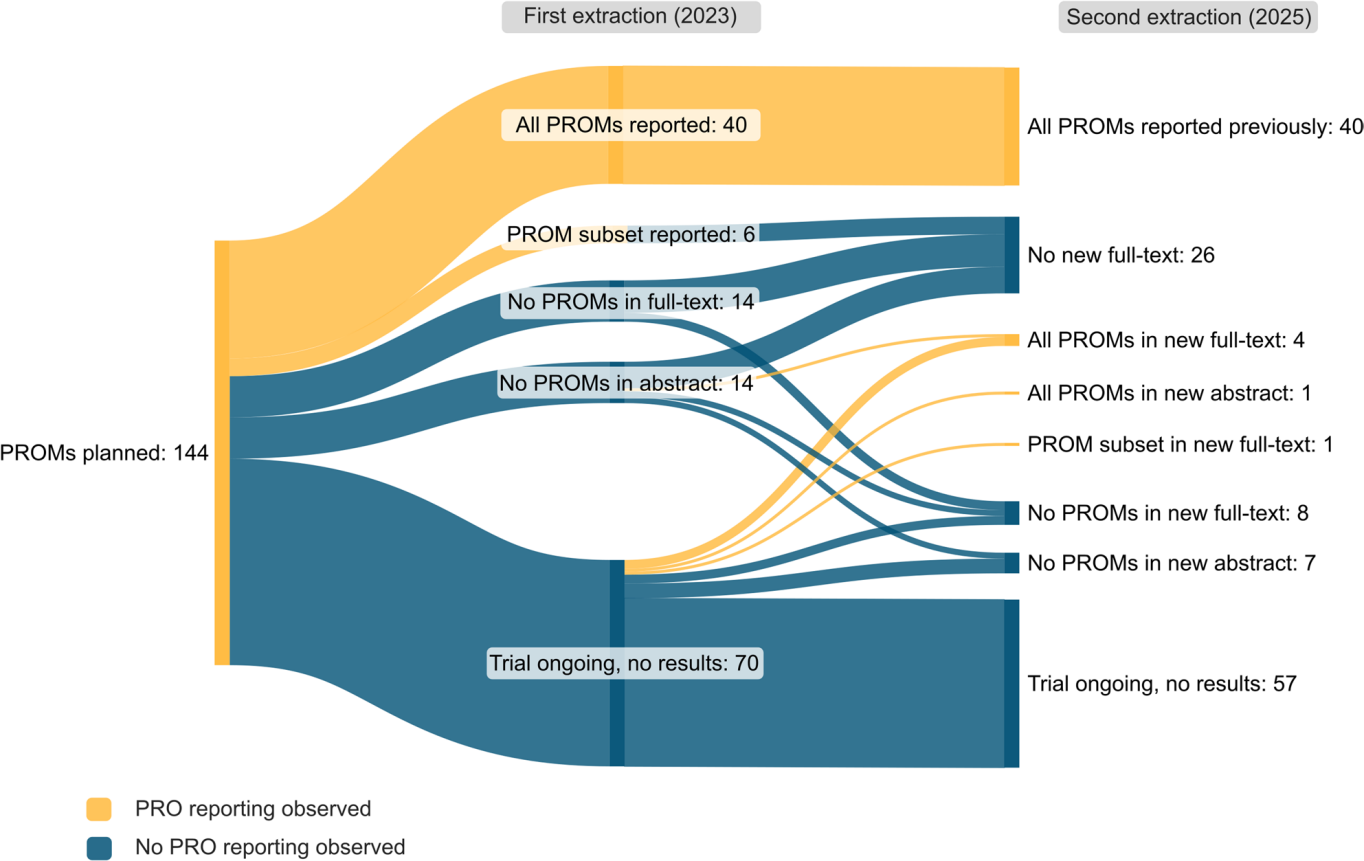
Sankey diagram displaying PRO reporting in NHL RCTs over a two-year period

Of the 487 initially identified RCTs, 144 RCTs (29.6%) planned to collect PROs as apparent from their protocol, registry entry, or trial publication. Many trials, which planned to collect PROs, were ongoing and initially presented no results (70 trials). Within two years, of these, only 13 trials published results: four RCTs consistently reported PROs in a new full-text or abstract, one RCT partially reported PROs in a new full-text, and three and five RCTs, respectively, entirely omitted PROs from their newly available full-text or abstract. While 40 RCTs that planned PRO assessment consistently reported results for all planned PROMs, six RCTs only partially reported PROMs in the first place. Trials which have failed to report results for the full set of PROMs did not address this gap within two years. Of the 14 trials entirely omitting PROs from the initially available full-text, 11 had no new full-text two years later, and three did not compensate for the omission in a subsequent full-text. Of the 14 trials entirely omitting PROs from their initially available abstract, nine had no new full-text, four did not report on PROs in a new full-text or abstract, and one consistently reported on PROs in a new full-text later on (Fig. 5).

PRO assessment was not planned in 136 RCTs (27.9%), i.e., PROs were not mentioned in their available full-text, and their protocol or registry entry, where available, did not suggest that PROs were intended to be included. Among trials that did not intend to integrate PROs, no trial was identified that nonetheless reported PROs.

For 207 RCTs (42.5%), it was not possible to determine whether PROs were planned to be evaluated, i.e., PROs were not mentioned in their protocol or registry entry, or neither was available, and no full-text could be located. Similar to trials in which PRO assessment was not planned, those lacking any information on PRO assessment also showed no PRO reporting at follow-up.

## Discussion

This scoping review identified 95 different PROMs referenced across clinical trial registry entries, study protocols, and publications in NHL RCTs, reflecting considerable heterogeneity in the instruments used. This variability points to a lack of standardization regarding which PROMs are most appropriate for capturing QoL in patients with NHL, raising concerns about the comparability, validity, and reliability of outcome assessments across trials. Although the body of literature on PROMs continues to grow, selecting instruments that are fit-for-purpose in RCTs remains a challenge, particularly given the proliferation of available measures and a rapidly evolving therapeutic landscape. Our review aimed not only to map the use of PROMs in NHL trials, but also to assess the consistency and trajectory of reporting. A notable finding of the present analysis is the substantial undercollection and underreporting of PROs in NHL RCTs. Of the 549 trials identified, 176 (32.1%) pre-specified PROs as outcomes. However, of those trials with published results, 25.0% of trials entirely and 10.5% partly omitted PRO findings from their main full-text publications, instead focusing solely on clinical outcomes, such as response and survival rates.

Our findings are consistent with prior studies that have identified underreporting of PROs in RCTs involving patients with hematologic malignancies [27–31]. The omission of PRO findings from trial publications is particularly concerning, given their essential role in reflecting the impact of NHL therapies, where chronic disease trajectories, symptom and side effect burdens, as well as survivorship considerations are pivotal to patient-centered care. Our data support the conclusions of Mercieca-Bebber and colleagues [32], whose systematic review appraised the use of the CONSORT PRO extension for assessing PRO reporting. The review found substantial omissions in the reporting of recommended CONSORT PRO items across many trials, potentially leading to misinterpretation of findings and consequently contributing to research waste [32]. The universal absence of references to the CONSORT PRO checklist among RCTs included in the present review underscores that methodological standards and reporting guidelines for PRO reporting remain neither widely disseminated nor prioritized in NHL RCTs.

A distinctive feature of this review is its systematic approach and longitudinal perspective on PRO reporting. By re-searching databases over a two-year period, we were able to assess whether initially unreported PROs were subsequently published. Incomplete or non-reporting of planned PROMs in the main full-text publications of studies was not addressed in subsequent publications within our observation period. Also, for trials in which PROMs were not intended, or their inclusion in the study design was unclear, no PRO reporting followed at later stages. These findings align with a recent systematic review on QoL endpoint collection, reporting, and framing in RCTs of indolent lymphomas, which noted that trials with improved QoL more often showed survival benefits, and suspected reluctance to analyze PROs if primary survival endpoints were not met. Additionally, this review’s authors noted a common practice of framing QoL results positively, even when results were neutral or negative [33]. It is conceivable that sometimes, a separate publication of PRO results was intended but never realized: perhaps due to resource constraints, competing priorities, or lacking capacity within the team to interpret and disseminate PRO findings. Another reason for reticence on the side of trial sponsors for reporting is the overuse of multiple legacy PROs in hematological malignancy trials, which are complicated to use and interpret, instead of using one sophisticated PRO instrument as a single measurement tool.

We analyzed sponsorship as reported in clinical trial registries and found that 34.1% of included trials were directly industry-sponsored. Notably, in our review, the absence of explicit industry-sponsoring did not preclude the involvement of a for-profit company as a collaborator providing funding or in-kind support. The proportion of industry-sponsored trials in our review was modest, likely reflecting our definition of trial sponsorship and the comprehensiveness of our literature and registry search, which captured a considerable number of smaller, investigator-initiated trials published in journals with a lower impact factor. 

The present analysis has limitations. In particular, trial registries were not subsequently re-searched. This pragmatic decision was based on the scoping design of the review and the specific objective of the re-searches, which prioritized identifying newly published PRO results rather than identifying entirely new trials. Recent ongoing registry-only trials, which could have contributed to the cross-sectional analysis of PROMs in NHL RCTs, may have been missed. Moreover, we did not apply the CONSORT PRO extension checklist to each trial that reported PROs. We therefore did not evaluate whether adherence to the CONSORT PRO extension was associated with higher reporting quality in this cohort. Such an analysis may be valuable, given that the reporting guideline is intended to enhance the completeness and transparency of PRO reporting [34], but adherence to the guideline was shown to be low, for example, in RCTs involving aggressive large B-cell lymphoma patients [35, 36].

Our analysis highlights a persistent gap in the dissemination of data reflecting the lived experiences of patients with NHL enrolled in RCTs. Addressing this shortfall will require not only more deliberate integration of PROs into trial design [11], but also stronger methodological rigor and accountability in their analysis and reporting. As our review focused exclusively on adults, and given similar concerns regarding the limited use of PROs in pediatric trials [37, 38], such research dedicated to children and adolescents is warranted. Future research should furthermore explore the use of PROs in routine clinical practice through naturalistic study designs, particularly employing instruments specifically developed for this population and setting [39, 40]. Guidance from the International Society for Quality of Life Research (ISOQOL) provides a framework for best practices in the implementation, interpretation, and integration of PROMs into routine care settings [41].

## Supplementary Information

Below is the link to the electronic supplementary material.

Supplementary Material 1

## Data Availability

No datasets were generated or analysed during the current study.
