# Supplementary material for: Use and reporting of patient-reported outcomes in randomized controlled trials in non-Hodgkin lymphoma: a scoping review
Source: J Patient Rep Outcomes. 2026 Jan 28;10:31. doi: 10.1186/s41687-026-00999-1 (PMC12923682; doi:10.1186/s41687-026-00999-1)
Supplement: Supplementary file 1 — Supplementary Material 1 [file 41687_2026_999_MOESM1_ESM.docx]

**Supplementary material**

Use and reporting of patient-reported outcomes in randomized controlled trials in non-Hodgkin lymphoma:
a scoping review

**Table of Contents**

[Appendix 1. Preferred Reporting Items for Systematic reviews and Meta-Analyses extension for Scoping Reviews (PRISMA-ScR) Checklist 2](#_Toc210487433)

[Appendix 2. Search strategies 4](#_Toc210487434)

[Appendix 3. List of 549 included randomized controlled trials 6](#_Toc210487435)

[Appendix 4. Use of patient-reported outcome measures (PROM) in included trials 20](#_Toc210487436)

# Appendix 1. Preferred Reporting Items for Systematic reviews and Meta-Analyses extension for Scoping Reviews (PRISMA-ScR) Checklist

| **SECTION** | **ITEM** | **PRISMA-ScR CHECKLIST ITEM** | **REPORTED** |
| --- | --- | --- | --- |
| **TITLE** | | | |
| Title | 1 | Identify the report as a scoping review. | X |
| **ABSTRACT** | | | |
| Structured summary | 2 | Provide a structured summary that includes (as applicable): background, objectives, eligibility criteria, sources of evidence, charting methods, results, and conclusions that relate to the review questions and objectives. | X |
| **INTRODUCTION** | | | |
| Rationale | 3 | Describe the rationale for the review in the context of what is already known. Explain why the review questions/objectives lend themselves to a scoping review approach. | X |
| Objectives | 4 | Provide an explicit statement of the questions and objectives being addressed with reference to their key elements (e.g., population or participants, concepts, and context) or other relevant key elements used to conceptualize the review questions and/or objectives. | X |
| **METHODS** | | | |
| Protocol and registration | 5 | Indicate whether a review protocol exists; state if and where it can be accessed (e.g., a Web address); and if available, provide registration information, including the registration number. | X |
| Eligibility criteria | 6 | Specify characteristics of the sources of evidence used as eligibility criteria (e.g., years considered, language, and publication status), and provide a rationale. | X |
| Information sources* | 7 | Describe all information sources in the search (e.g., databases with dates of coverage and contact with authors to identify additional sources), as well as the date the most recent search was executed. | X |
| Search | 8 | Present the full electronic search strategy for at least 1 database, including any limits used, such that it could be repeated. | X  (in the appendix) |
| Selection of sources of evidence† | 9 | State the process for selecting sources of evidence (i.e., screening and eligibility) included in the scoping review. | X |
| Data charting process‡ | 10 | Describe the methods of charting data from the included sources of evidence (e.g., calibrated forms or forms that have been tested by the team before their use, and whether data charting was done independently or in duplicate) and any processes for obtaining and confirming data from investigators. | X |
| Data items | 11 | List and define all variables for which data were sought and any assumptions and simplifications made. | X |
| Critical appraisal of individual sources of evidence§ | 12 | If done, provide a rationale for conducting a critical appraisal of included sources of evidence; describe the methods used and how this information was used in any data synthesis (if appropriate). | Not applicable |
| Synthesis of results | 13 | Describe the methods of handling and summarizing the data that were charted. | X |
| **RESULTS** | | | |
| Selection of sources of evidence | 14 | Give numbers of sources of evidence screened, assessed for eligibility, and included in the review, with reasons for exclusions at each stage, ideally using a flow diagram. | X |
| Characteristics of sources of evidence | 15 | For each source of evidence, present characteristics for which data were charted and provide the citations. | X  (citations in the appendix) |
| Critical appraisal within sources of evidence | 16 | If done, present data on critical appraisal of included sources of evidence (see item 12). | Not applicable |
| Results of individual sources of evidence | 17 | For each included source of evidence, present the relevant data that were charted that relate to the review questions and objectives. | X |
| Synthesis of results | 18 | Summarize and/or present the charting results as they relate to the review questions and objectives. | X |
| **DISCUSSION** | | | |
| Summary of evidence | 19 | Summarize the main results (including an overview of concepts, themes, and types of evidence available), link to the review questions and objectives, and consider the relevance to key groups. | X |
| Limitations | 20 | Discuss the limitations of the scoping review process. | X |
| Conclusions | 21 | Provide a general interpretation of the results with respect to the review questions and objectives, as well as potential implications and/or next steps. | X |
| **FUNDING** | | | |
| Funding | 22 | Describe sources of funding for the included sources of evidence, as well as sources of funding for the scoping review. Describe the role of the funders of the scoping review. | X |

JBI = Joanna Briggs Institute; PRISMA-ScR = Preferred Reporting Items for Systematic reviews and Meta-Analyses extension for Scoping Reviews.

* Where *sources of evidence* (see second footnote) are compiled from, such as bibliographic databases, social media platforms, and Web sites.

† A more inclusive/heterogeneous term used to account for the different types of evidence or data sources (e.g., quantitative and/or qualitative research, expert opinion, and policy documents) that may be eligible in a scoping review as opposed to only studies. This is not to be confused with *information sources* (see first footnote).

‡ The frameworks by Arksey and O’Malley (6) and Levac and colleagues (7) and the JBI guidance (4, 5) refer to the process of data extraction in a scoping review as data charting*.*

§ The process of systematically examining research evidence to assess its validity, results, and relevance before using it to inform a decision. This term is used for items 12 and 19 instead of "risk of bias" (which is more applicable to systematic reviews of interventions) to include and acknowledge the various sources of evidence that may be used in a scoping review (e.g., quantitative and/or qualitative research, expert opinion, and policy document).

*From:* Tricco AC, Lillie E, Zarin W, O'Brien KK, Colquhoun H, Levac D, et al. PRISMA Extension for Scoping Reviews (PRISMAScR): Checklist and Explanation. Ann Intern Med. 2018;169:467–473.
[doi: 10.7326/M18-0850](http://annals.org/aim/fullarticle/2700389/prisma-extension-scoping-reviews-prisma-scr-checklist-explanation).

# Appendix 2. Search strategies

**Search strategy for initial search, 1 January 2017 to 19 January 2023**

**Ovid MEDLINE**

| **#** | **Search** | **Results** |
| --- | --- | --- |
| 1 | *lymphoma/ | 37586 |
| 2 | lymphom*.tw,kf. | 204145 |
| 3 | ((high grade* adj2 (malignanc* or lymphom*)) or HGBCL).tw,kf. | 2928 |
| 4 | *Lymphoma, B-Cell/ | 12681 |
| 5 | exp Lymphoma, Non-Hodgkin/ | 111874 |
| 6 | (non-hodgkin* or non hodgkin* or nonhodgkin* or no hodgkin* or nhl).tw,kf. | 43691 |
| 7 | (reticulosarcoma* or reticulum cell sarcom* or (lymphatic adj2 sarcom*) or lymphosarcom*).tw,kf. | 7528 |
| 8 | or/1-7 | 240331 |
| 9 | randomized controlled trial.pt. | 584761 |
| 10 | controlled clinical trial.pt. | 95156 |
| 11 | randomi?ed.ab. | 704657 |
| 12 | placebo.ab. | 235080 |
| 13 | clinical trials as topic.sh. | 200770 |
| 14 | randomly.ab. | 400229 |
| 15 | trial.ti. | 277925 |
| 16 | or/9-15 | 1543317 |
| 17 | exp animals/ not humans/ | 5084212 |
| 18 | 16 not 17 | 1422303 |
| 19 | clinical trial, phase iii/ | 21283 |
| 20 | ("Phase 3" or "phase3" or "phase III" or P3 or "PIII").ti,ab,kw. | 80794 |
| 21 | (19 or 20) not 17 | 79958 |
| 22 | 18 or 21 | 1461778 |
| 23 | 8 and 22 | 9725 |
| 24 | limit 23 to yr="2017 -Current" | 2567 |
| 25 | hodgkin disease/ not Lymphoma, Non-Hodgkin/ | 30691 |
| 26 | 24 not 25 | 2378 |

**Cochrane Central Register of Controlled Trials (CENTRAL)**

| **#** | **Search** |
| --- | --- |
| 1 | MeSH descriptor: [Lymphoma] this term only |
| 2 | lymphom*:TI,AB,KW |
| 3 | ((high grade* NEAR/2 (malignanc* OR lymphom*)) OR HGBCL):TI,AB,KW |
| 4 | MeSH descriptor: [Lymphoma, B-Cell] this term only |
| 5 | MeSH descriptor: [Lymphoma, Non-Hodgkin] this term only |
| 6 | (non-hodgkin* or non hodgkin* or nonhodgkin* or no hodgkin* or nhl):TI,AB,KW |
| 7 | (reticulosarcoma* or reticulum cell sarcom* or (lymphatic NEAR/2 sarcom*) or lymphosarcom*):TI,AB,KW |
| 8 | #1 OR #2 OR #3 OR #4 OR #5 OR #6 OR #7 with Publication Year from 2017 to 2023, in Trials |
| 9 | MeSH descriptor: [Hodgkin Disease] this term only |
| 10 | #9 NOT #5 with Publication Year from 2017 to 2023, in Trials |
| 11 | #8 NOT #10 |

**ClinicalTrials.gov**

Interventional Studies | Lymphoma OR "non-hodgkin" OR "non hodgkin" OR nonhodgkin OR "no hodgkin" OR nhl OR reticulosarcoma OR "reticulum cell sarcoma" OR "lymphatic sarcoma" OR lymphosarcoma

Restrict to: 01/2017 to 01/2023

**WHO International Clinical Trials Registry Platform (ICTRP)**

Lymphoma OR "non-hodgkin" OR "non hodgkin" OR nonhodgkin OR "no hodgkin" OR nhl OR reticulosarcoma OR "reticulum cell sarcoma" OR "lymphatic sarcoma" OR lymphosarcoma

Restrict to: 01/2017 to 01/2023

**Search strategy for subsequent searches, 20 January 2023 to 4 February 2025**

**OVID MEDLINE**

| **#** | **Search** |
| --- | --- |
| 1 | *lymphoma/ |
| 2 | lymphom*.tw,kf. |
| 3 | ((high grade* adj2 (malignanc* or lymphom*)) or HGBCL).tw,kf. |
| 4 | *Lymphoma, B-Cell/ |
| 5 | exp Lymphoma, Non-Hodgkin/ |
| 6 | (non-hodgkin* or non hodgkin* or nonhodgkin* or no hodgkin* or nhl).tw,kf. |
| 7 | (reticulosarcoma* or reticulum cell sarcom* or (lymphatic adj2 sarcom*) or lymphosarcom*).tw,kf. |
| 8 | or/1-7 |
| 9 | randomized controlled trial.pt. |
| 10 | controlled clinical trial.pt. |
| 11 | randomi?ed.ab. |
| 12 | placebo.ab. |
| 13 | clinical trials as topic.sh. |
| 14 | randomly.ab. |
| 15 | trial.ti. |
| 16 | or/9-15 |
| 17 | exp animals/ not humans/ |
| 18 | 16 not 17 |
| 19 | clinical trial, phase iii/ |
| 20 | ("Phase 3" or "phase3" or "phase III" or P3 or "PIII").ti,ab,kw. |
| 21 | (19 or 20) not 17 |
| 22 | 18 or 21 |
| 23 | 8 and 22 |
| 24 | limit 23 to yr="2017 -Current" |
| 25 | hodgkin disease/ not Lymphoma, Non-Hodgkin/ |
| 26 | 24 not 25 |

**CENTRAL**

| **#** | **Search** |
| --- | --- |
| 1 | MeSH descriptor: [Lymphoma] this term only |
| 2 | lymphom*:TI,AB,KW |
| 3 | ((high grade* NEAR/2 (malignanc* OR lymphom*)) OR HGBCL):TI,AB,KW |
| 4 | MeSH descriptor: [Lymphoma, B-Cell] this term only |
| 5 | MeSH descriptor: [Lymphoma, Non-Hodgkin] this term only |
| 6 | (non-hodgkin* or non hodgkin* or nonhodgkin* or no hodgkin* or nhl):TI,AB,KW |
| 7 | (reticulosarcoma* or reticulum cell sarcom* or (lymphatic NEAR/2 sarcom*) or lymphosarcom*):TI,AB,KW |
| 8 | #1 OR #2 OR #3 OR #4 OR #5 OR #6 OR #7 with Publication Year from 2017 to 2025, in Trials |
| 9 | MeSH descriptor: [Hodgkin Disease] this term only |
| 10 | #9 NOT #5 with Publication Year from 2017 to 2025, in Trials |
| 11 | #8 NOT #10 |

| Appendix 3. List of 549 included randomized controlled trials | | | | | |
| --- | --- | --- | --- | --- | --- |
| **#** | **Study name** (acronym or first author/year) | **Registration number** (NCT, EudraCT, CTRI, …) | **Source(s)** (DOI, registration number) | **Identified by 2025 search** |  |
| 1 | – | 2015-002693-20 | 10.1200/JCO.2019.37.15_suppl.e19065 |  |  |
| 2 | – | ChiCTR1800015137 | ChiCTR1800015137 |  |  |
| 3 | – | ChiCTR1800016003 | ChiCTR1800016003 |  |  |
| 4 | – | ChiCTR1800016732 | ChiCTR1800016732 |  |  |
| 5 | – | ChiCTR1800018708 | ChiCTR1800018708 |  |  |
| 6 | – | ChiCTR1800018720 | ChiCTR1800018720 |  |  |
| 7 | – | ChiCTR1800017310 | ChiCTR1800017310 |  |  |
| 8 | – | ChiCTR1800018734 | ChiCTR1800018734 |  |  |
| 9 | – | ChiCTR2000035488 | ChiCTR2000035488 |  |  |
| 10 | – | ChiCTR1900022909 | ChiCTR1900022909 |  |  |
| 11 | – | ChiCTR2000037503 | ChiCTR2000037503 |  |  |
| 12 | – | ChiCTR1900028209 | ChiCTR1900028209 |  |  |
| 13 | – | ChiCTR2000032550 | 10.1182/blood-2021-146338 |  |  |
| 14 | – | CTRI/2016/05/006904 | 10.4103/ijc.IJC_633_20, 10.1200/JCO.2018.36.15_suppl.e19552 |  |  |
| 15 | – | ChiCTR2000034753 | ChiCTR2000034753 |  |  |
| 16 | – | ChiCTR1900025028 | ChiCTR1900025028 |  |  |
| 17 | – | ChiCTR2000035106 | ChiCTR2000035106 |  |  |
| 18 | – | ChiCTR2000035401 | ChiCTR2000035401 |  |  |
| 19 | – | ChiCTR2000037570 | ChiCTR2000037570 |  |  |
| 20 | – | ChiCTR2000040602 | 10.1136/jitc-2024-008895 |  |  |
| 21 | – | ChiCTR2100041594 | ChiCTR2100041594 |  |  |
| 22 | – | ChiCTR2100041905 | ChiCTR2100041905 |  |  |
| 23 | – | ChiCTR2100042685 | ChiCTR2100042685 |  |  |
| 24 | – | ChiCTR2100048196 | ChiCTR2100048196 |  |  |
| 25 | – | ChiCTR2100054354 | ChiCTR2100054354 |  |  |
| 26 | – | ChiCTR-IIR-17010787 | 10.2147/CMAR.S219242 |  |  |
| 27 | – | CTRI/2012/11/003129 | 10.1200/JCO.2017.35.15_suppl.7550, 10.1200/JGO.19.00248 |  |  |
| 28 | – | CTRI/2017/08/009336 | 10.4103/ijoy.ijoy_126_24 |  |  |
| 29 | – | CTRI/2018/07/014885 | 10.1007/s00280-023-04530-x |  |  |
| 30 | – | ChiCTR2000031889 | ChiCTR2000031889 |  |  |
| 31 | – | NCT00006250 | 10.1111/bjh.16555, 10.1182/blood.V108.11.534.534 |  |  |
| 32 | – | CTRI/2020/10/028220 | CTRI/2020/10/028220 |  |  |
| 33 | – | CTRI/2022/11/047204 | CTRI/2022/11/047204 |  |  |
| 34 | – | ChiCTR2100043614 | ChiCTR2100043614 |  |  |
| 35 | – | IRCT20191023045208N1 | 10.7754/Clin.Lab.2022.220317 |  |  |
| 36 | – | IRCT20200128046292N1 | 10.1007/s00262-021-02889-5 |  |  |
| 37 | – | ChiCTR-IIR-17014083 | ChiCTR-IIR-17014083 |  |  |
| 38 | – | jRCT2073210013 | jRCT2073210013 |  |  |
| 39 | – | ISRCTN77237304 | 10.2298/AOO231207003N, 10.1200/JCO.2022.40.16_suppl.e19579, 10.31557/APJCP.2024.25.4.1315 |  |  |
| 40 | – | jRCT2031200383 | jRCT2031200383 |  |  |
| 41 | – | KCT0007009 | KCT0007009 |  |  |
| 42 | – | NCT00472056 | 10.1111/bjh.14731 |  |  |
| 43 | – | NCT00765245 | 10.1038/leu.2016.255 |  |  |
| 44 | – | NCT00931918 | 10.1200/JCO.2017.73.2784 |  |  |
| 45 | – | NCT01180049 | 10.1080/10428194.2017.1357175 |  |  |
| 46 | – | NCT01197560 | 10.1158/1078-0432.CCR-16-2818 |  |  |
| 47 | – | NCT01232556 | 10.1111/bjh.14820 |  |  |
| 48 | – | NCT01501136 | 10.1002/ijc.33329 |  |  |
| 49 | – | NCT01691807 | 10.1002/cpdd.1133 |  |  |
| 50 | – | NCT01501149 | 10.1182/blood-2019-127811, 10.1001/jamaoncol.2022.1968 |  |  |
| 51 | – | NCT01625455 | 10.1001/jamadermatol.2018.2510 |  |  |
| 52 | – | NCT01960192 | 10.1007/s11060-018-2970-x |  |  |
| 53 | – | NCT01664975 | 10.1111/bjh.14763,  10.1177/1758835920923829 |  |  |
| 54 | – | ChiCTR1800015922 | ChiCTR1800015922 |  |  |
| 55 | – | NCT02501473 | 10.1080/10428194.2021.2010057 |  |  |
| 56 | – | ChiCTR1800019725 | ChiCTR1800019725 |  |  |
| 57 | – | NCT02213861 | 10.1016/j.jid.2018.03.615 |  |  |
| 58 | – | NCT02983942 | NCT02983942 |  |  |
| 59 | – | NCT02301494 | NCT02301494 |  |  |
| 60 | – | ChiCTR1900022510 | ChiCTR1900022510 |  |  |
| 61 | – | ChiCTR2000035631 | ChiCTR2000035631 |  |  |
| 62 | – | NCT03151044 | NCT03151044 |  |  |
| 63 | – | NCT03188198 | NCT03188198 |  |  |
| 64 | – | ChiCTR2000036602 | ChiCTR2000036602 |  |  |
| 65 | – | CTR20161069 | 10.3389/fmed.2021.609116,  10.1182/blood-2019-125456 |  |  |
| 66 | – | NCT03229616 | NCT03229616 |  |  |
| 67 | – | NCT02533700 | 10.1186/s13073-020-00739-0 |  |  |
| 68 | – | IRCT20120109008665N8 | IRCT20120109008665N8 |  |  |
| 69 | – | NCT02631239 | 10.1016/j.ijrobp.2024.07.1457, 10.1016/j.xinn.2023.100426 |  |  |
| 70 | – | NCT02811783 | NCT02811783 |  |  |
| 71 | – | NCT03309878 | 10.1200/JCO.2020.38.15_suppl.TPS8072 |  |  |
| 72 | – | IRCT20130616013690N6 | IRCT20130616013690N6 |  |  |
| 73 | – | jRCTs051180026 | 10.2169/internalmedicine.1314-22 |  |  |
| 74 | – | NCT02943642 | NCT02943642 |  |  |
| 75 | – | NCT03318835 | NCT03318835 |  |  |
| 76 | – | NCT03011814 | 10.1182/blood-2023-188053 |  |  |
| 77 | – | NCT03023358 | NCT03023358 |  |  |
| 78 | – | NCT00352846 | 10.1016/j.clml.2012.11.002 |  |  |
| 79 | – | NCT00566228 | 10.1016/j.bbmt.2016.01.024,  10.1182/blood-2021-148454 |  |  |
| 80 | – | NCT03479918 | NCT03479918 |  |  |
| 81 | – | NCT03292406 | NCT03292406 |  |  |
| 82 | – | NCT04212013 | 10.1200/JCO.2021.39.15_suppl.TPS7576 |  |  |
| 83 | – | NCT03355768 | NCT03355768 |  |  |
| 84 | – | NCT03579082 | 10.3389/fonc.2021.687374 |  |  |
| 85 | – | NCT03647072 | 10.1007/s12032-020-01452-z |  |  |
| 86 | – | NCT03650933 | NCT03650933 |  |  |
| 87 | – | NCT03670901 | 2018-002342-36 |  |  |
| 88 | – | NCT01164475 | 10.1038/s41409-018-0253-y |  |  |
| 89 | – | NCT01483664 | 10.1002/pon.5371,  10.1002/cncr.31767 |  |  |
| 90 | – | NCT03777085 | NCT03777085 |  |  |
| 91 | – | NCT04083066 | NCT04083066 |  |  |
| 92 | – | NCT04129710 | NCT04129710 |  |  |
| 93 | – | NCT03454945 | 10.1080/09546634.2019.1667474 |  |  |
| 94 | – | NCT04152577 | NCT04152577 |  |  |
| 95 | – | NCT04231448 | NCT04231448 |  |  |
| 96 | – | NCT01729806 | 10.1158/1078-0432.CCR-19-0438, 10.1182/blood.V130.Suppl_1.4086.4086 |  |  |
| 97 | – | NCT01765231 | 10.1016/j.clml.2018.11.008 |  |  |
| 98 | – | NCT01767714 | 10.1111/trf.14426 |  |  |
| 99 | – | NCT01870479 | 10.1007/s00520-019-04666-8 |  |  |
| 100 | – | NCT04236141 | 10.4103/jcrt.jcrt_269_24 |  |  |
| 101 | – | NCT03631862 | NCT03631862 |  |  |
| 102 | – | NCT02221492 | 10.1007/s12185-018-2505-4 |  |  |
| 103 | – | NCT04481815 | NCT04481815 |  |  |
| 104 | – | NCT04553393 | NCT04553393 |  |  |
| 105 | – | NCT04365036 | NCT04365036 |  |  |
| 106 | – | NCT03014102 | 10.1016/j.cdtm.2021.05.003 |  |  |
| 107 | – | NCT03100175 | NCT03100175 |  |  |
| 108 | – | NCT04914143 | NCT04914143 |  |  |
| 109 | – | NCT03151057 | NCT03151057 |  |  |
| 110 | – | NCT04668690 | NCT04668690 |  |  |
| 111 | – | NCT03417765 | NCT03417765 |  |  |
| 112 | – | NCT03456466 | NCT03456466 |  |  |
| 113 | – | NCT03505762 | NCT03505762 |  |  |
| 114 | – | NCT03670888 | NCT03670888 |  |  |
| 115 | – | NCT04922567 | NCT04922567 |  |  |
| 116 | – | NCT05245656 | NCT05245656 |  |  |
| 117 | – | NCT03980379 | NCT03980379 |  |  |
| 118 | – | NCT05274139 | NCT05274139 |  |  |
| 119 | – | NCT05280626 | NCT05280626 |  |  |
| 120 | – | NCT05239910 | NCT05239910 |  |  |
| 121 | – | NCT04134247 | NCT04134247 |  |  |
| 122 | – | NCT04701554 | 10.3390/jcm11123421 |  |  |
| 123 | – | NCT04948788 | NCT04948788 |  |  |
| 124 | – | NCT05110742 | NCT05110742 |  |  |
| 125 | – | NCT05351346 | NCT05351346 |  |  |
| 126 | – | NCT05376709 | NCT05376709 |  |  |
| 127 | – | NCT05604417 | NCT05604417 |  |  |
| 128 | – | NCT05507541 | NCT05507541 |  |  |
| 129 | – | NCT05626322 | 10.1182/blood-2023-181202 |  |  |
| 130 | – | NCT05370547 | NCT05370547 |  |  |
| 131 | – | TCTR20180202004 | 10.1097/01.HS9.0000565684.68271.82 |  |  |
| 132 | – | RBR-544pfq | https://ensaiosclinicos.gov.br/rg/RBR-544pfq |  |  |
| 133 | – | TCTR20191025002 | TCTR20191025002 |  |  |
| 134 | – | UMIN000026758 | UMIN000026758 |  |  |
| 135 | – | UMIN000034569 | UMIN000034569 |  |  |
| 136 | – | UMIN000007283 | 10.1002/hon.2524 |  |  |
| 137 | – | jRCTs051220169 | 10.2196/54882 | yes |  |
| 138 | – | NCT02752815 | 10.1002/cac2.12462 | yes |  |
| 139 | – | NCT06190301 | 10.1136/bmjopen-2024-084904 | yes |  |
| 140 | – | ChiCTR2300070327 | 10.3892/ol.2023.13844 | yes |  |
| 141 | – | NCT02323659 | 10.5114/ada.2021.106206 | yes |  |
| 142 | – | ACTRN12623000705684 | 10.1136/bmjopen-2023-081084 | yes |  |
| 143 | – | NCT05896163 | 10.1182/blood-2023-182275 | yes |  |
| 144 | – | 2021-001937-38  (non-traceable) | 10.1007/s00432-022-04324-3 | yes |  |
| 145 | A051301, BMT-CTN 1201, IRONCLAD | NCT02443077 | 10.1200/JCO.2017.35.15_suppl.TPS7566, 10.1182/blood-2023-173742 |  |  |
| 146 | AATT | NCT00984412  (does not match study) | 10.1200/JCO.24.00554, 10.1182/blood.2020008825 |  |  |
| 147 | AC-CHOP Study | NCT05678933 | NCT05678933 |  |  |
| 148 | ACCRU-LY-2001 | NCT04665115 | NCT04665115 |  |  |
| 149 | ACE-LY-003 | NCT02180711 | 10.1111/bjh.19787, 10.1200/JCO.2018.36.15_suppl.7549, 10.1097/HS9.0000000000000060 |  |  |
| 150 | ACE-LY-110 | NCT03205046 | 10.1080/10428194.2021.1938027 |  |  |
| 151 | ACT-1 | NCT00646854 | 10.1182/blood-2018-99-110429 |  |  |
| 152 | AFL trial | – | 10.1080/10428194.2023.2295792 | yes |  |
| 153 | Ai 2019 | – | 10.13201/j.issn.1001-1781.2019.10.022 |  |  |
| 154 | Alagizy 2023,  Hegazy 2024 | – | 10.31557/APJCP.2024.25.7.2351,  10.1016/S2152-2650(23)01283-1 | yes |  |
| 155 | ALCANZA | NCT01578499 | 10.1016/j.ejca.2020.04.010,  10.1016/S0140-6736(17)31266-7, 10.1016/j.jval.2019.09.664 |  |  |
| 156 | Alliance A051701 | NCT03984448 | 10.1182/blood-2021-151266 |  |  |
| 157 | Alliance A059102 | NCT04803201 | 10.1200/JCO.2022.40.16_suppl.TPS7593 |  |  |
| 158 | Alliance CALGB 50303 | NCT00118209 | 10.1200/JCO.18.01994 |  |  |
| 159 | Alliance CALGB 50403 | NCT00310037 | 10.1002/ajh.25783 |  |  |
| 160 | Alliance CALGB 50901 | NCT01190449 | 10.1111/bjh.15768 |  |  |
| 161 | Alliance CALGB 50904 | NCT01286272 | 10.1002/cncr.32289 |  |  |
| 162 | Alliance CALGB 51101 | NCT01511562 | 10.1200/JCO.2021.39.15_suppl.7506 |  |  |
| 163 | ALPHA-3 | NCT06500273 | 10.1182/blood-2024-194909 | yes |  |
| 164 | AMAFRICA | – | 10.1186/s12885-019-6478-3 |  |  |
| 165 | AMC-075 | NCT01193842 | 10.1182/blood.2019003959,  10.1182/blood-2019-129687 |  |  |
| 166 | Angsutararux 2023 | – | 10.1093/ofid/ofad500.852 | yes |  |
| 167 | ANHL-1931 | NCT04759586 | NCT04759586 |  |  |
| 168 | ARCHED,  GLA 2022-1 | NCT05820841 | 10.1159/000540557 | yes |  |
| 169 | ARGO | NCT03422523 | NCT03422523 |  |  |
| 170 | ASCT-001 | NCT04880746 | NCT04880746 |  |  |
| 171 | ASPEN | NCT03053440 | 10.1080/14796694.2024.2355079,  10.2217/fon-2018-0163 |  |  |
| 172 | ASSIST-FL | NCT01419665 | 10.1093/annonc/mdx373,  10.1016/S2352-3026(17)30106-0 |  |  |
| 173 | ASTX660-03 | NCT05403450 | https://astx.com/wp-content/uploads/2022/06/2022_ASTX660_Poster_TCLF_abst-N-009_Poligone_final.pdf |  |  |
| 174 | AUGMENT | NCT01938001 | 10.1200/JCO.19.00010 |  |  |
| 175 | Aviles 2019 (a) | – | 10.1002/pro6.1070 |  |  |
| 176 | Aviles 2019 (b) | – | 10.1002/pro6.1071 |  |  |
| 177 | AVOID neutropenia | NCT02044276 | 10.1007/s00520-020-05711-7 |  |  |
| 178 | B-MIND | NCT02763319 | NCT02763319 |  |  |
| 179 | B-NHL002 | NCT05164770 | NCT05164770 |  |  |
| 180 | BAL | NCT03187210 | NCT03187210 |  |  |
| 181 | BDH-WM01 | NCT02844322 | 10.1002/hon.3165_539,  10.1182/blood-2019-130133 |  |  |
| 182 | BEB trial | NCT02278796 | 10.1182/blood-2022-157917 | yes |  |
| 183 | BELIEVE-01 | NCT05234684 | 10.1182/blood-2022-158865 |  |  |
| 184 | BELINDA | NCT03570892 | 10.1056/NEJMoa2116596 |  |  |
| 185 | BIORIX | NCT01701232 | 10.1002/hon.2693 |  |  |
| 186 | BLOCAGE-01 | NCT02313389 | NCT02313389 | yes |  |
| 187 | Bock 2024 | NCT06238648 | 10.1200/JCO.2024.42.16_suppl.TPS7100 | yes |  |
| 188 | BRCAP-GELTAMO12 | NCT01848132 | 10.1182/blood.V128.22.4201.4201, 10.1002/hon.2438_49 |  |  |
| 189 | BRIGHT | NCT00877006 | 10.1016/j.clml.2016.01.001,  10.1182/blood-2013-11-531327, 10.1200/JCO.18.00605 |  |  |
| 190 | BRUIN MCL-321 | NCT04662255 | 10.1200/JCO.2023.41.16_suppl.TPS7587, 10.2217/fon-2022-0976 |  |  |
| 191 | C-Little | jRCTs041180094 | 10.1007/s13555-021-00655-0,  10.1007/s40268-024-00465-7 |  |  |
| 192 | CAMS lymphoma | NCT04511351 | 10.1016/j.ijrobp.2022.04.001 |  |  |
| 193 | CAVALLI | NCT02055820 | 10.1182/blood.2020006578 |  |  |
| 194 | CC-220-DLBCL-001 | NCT04884035 | 10.1016/S2152-2650(24)01534-9 | yes |  |
| 195 | CELESTIMO,  GO42909 | NCT04712097 | 10.1200/JCO.2022.40.16_suppl.TPS7588 |  |  |
| 196 | Chaturverdi 2023 | – | 10.1158/1538-7445.AM2023-CT082 | yes |  |
| 197 | CHEMO-T | NCT01719835 | 10.1016/S2352-3026(18)30039-5 |  |  |
| 198 | ChiCGB 2.0 | NCT05466318 | NCT05466318 |  |  |
| 199 | CHRONOS-3 | NCT02367040 | 10.1016/S1470-2045(21)00145-5 |  |  |
| 200 | CHRONOS-4 | NCT02626455 | 10.1182/bloodadvances.2024013236 | yes |  |
| 201 | Chuang 2017 | – | 10.1016/j.ijnurstu.2017.01.004 |  |  |
| 202 | CIBI110B201 | NCT05039658 | NCT05039658 |  |  |
| 203 | CIBI301A201 | NCT02945215 | 10.1038/s41598-020-68360-0 |  |  |
| 204 | CIBI301A301 | NCT02867566 | 10.1007/s12325-020-01603-8 |  |  |
| 205 | CISL 12-05 | NCT03794167 | 10.4143/crt.2022.004 |  |  |
| 206 | CITADEL-302 | NCT04796922 | NCT04796922 |  |  |
| 207 | CITADEL-310 | NCT04849715 | NCT04849715 |  |  |
| 208 | COALITION | NCT04914741 | NCT04914741 |  |  |
| 209 | COASTAL | NCT04745832 | 10.1182/blood-2021-148015 |  |  |
| 210 | COMPLEMENT  A + B | NCT01077518 | 10.1111/bjh.17420 |  |  |
| 211 | CONTRALTO | NCT02187861 | 10.1182/blood-2018-99-116806, 10.1182/blood.2020005588 |  |  |
| 212 | CSIIT-T13 | NCT03617432 | NCT03617432 |  |  |
| 213 | CSPC-DMS-DLBCL-02 | NCT03022123 | NCT03022123 |  |  |
| 214 | CSPC-DMS-LY-02 | NCT03952572 | NCT03952572 |  |  |
| 215 | CSWOG0001 | NCT01793844 | 10.4143/crt.2018.230 |  |  |
| 216 | CT-P10 3.4 | NCT02260804 | 10.1016/j.clml.2021.08.005 |  |  |
| 217 | CT-P10-3.3 | NCT02162771 | 10.1016/S2352-3026(17)30120-5, 10.1182/bloodadvances.2021004484 |  |  |
| 218 | CX-659-401 | NCT05319028 | 10.1182/blood-2022-165502 |  |  |
| 219 | CZAR-1 | NCT04263480 | NCT04263480 |  |  |
| 220 | D-CHOP | NCT03553537 | NCT03553537 |  |  |
| 221 | D-GEMOX | KCT0004590 | KCT0004590 |  |  |
| 222 | DALY 2 EU | NCT04844866 | 10.1097/01.HS9.0000852292.38263.b8 |  |  |
| 223 | DENOSULY | UMIN000038881 | UMIN000038881 |  |  |
| 224 | DIAL | NCT03038672 | 10.1182/blood-2019-130449, 10.1200/JCO.2022.40.17_suppl.LBA7564 |  |  |
| 225 | DLBCL-005 | NCT03213977 | NCT03213977 |  |  |
| 226 | DLBCL-2015 | NCT02842931 | 10.17650/1818-8346-2021-16-3-86-94, 10.1016/S2152-2650(22)01497-5 |  |  |
| 227 | DOBL | NCT02964858 | 10.1002/cnr2.1161 |  |  |
| 228 | Dong 2024 | – | 10.13422/j.cnki.syfjx.20241995 | yes |  |
| 229 | DSHNHL2006-1B,  ACT-2 | NCT00725231 | 10.1038/s41375-020-0838-5,  EudraCT 2007-000821-23 |  |  |
| 230 | DURABILITY | NCT03161223 | NCT03161223 |  |  |
| 231 | E-CELERATE | NCT05181540 | NCT05181540 |  |  |
| 232 | E-SCHOLAR | CTRI/2020/06/025818 | CTRI/2020/06/025818 |  |  |
| 233 | EA4151 | NCT03267433 | 10.1182/blood-2024-212973 |  |  |
| 234 | ECHELON-2 | NCT01777152 | 10.1182/blood-2020-134398, 10.1016/S0140-6736(18)32984-2 |  |  |
| 235 | ECHELON-3,  SGN35-031 | NCT04404283 | 10.1200/JCO-24-02242,  10.1182/blood-2021-151583 |  |  |
| 236 | ECHO,  ACE-LY-308 | NCT02972840 | 10.1002/hon.2632,  https://library.ehaweb.org/eha/2024/  eha2024-congress/document?c_id=  4136515&cm_ii=443951&type=document443951 |  |  |
| 237 | ECOG-ACRIN E1411 | NCT01415752 | 10.1182/blood.2024023962, 10.1182/blood-2022-170381 |  |  |
| 238 | ECOG-ACRIN E1412 | NCT01856192 | NCT01856192 |  |  |
| 239 | ECOG-ACRIN E2408, BIONIC | NCT01216683 | 10.1182/blood-2020-140502 |  |  |
| 240 | ECOG-ACRIN EA4181 | NCT04115631 | NCT04115631 |  |  |
| 241 | ECOG-AMC34 | NCT00049036 | 10.3324/haematol.2019.243386 |  |  |
| 242 | ECOG-E4402,  RESORT | NCT00075946 | 10.1200/JCO.23.01912, 10.1200/JCO.2014.57.6801 |  |  |
| 243 | ECWM-1 | NCT01788020 | 10.1200/JCO.22.01805 |  |  |
| 244 | EMILY | NCT05298293 | NCT05298293 |  |  |
| 245 | ENGAGE | ACTRN12617000068369 | 10.1016/j.soncn.2024.151592 |  |  |
| 246 | ENGINE | NCT03263026 | 10.2217/fon-2020-0176 |  |  |
| 247 | ENRICH | ISRCTN11038174 | 10.1182/blood-2024-199710,  EudraCT 2015-000832-13 |  |  |
| 248 | EnTe-HBV | NCT04539119 | NCT04539119 |  |  |
| 249 | EORTC 20971-22997 | NCT00014326 | 10.1016/S0167-8140(21)00398-4, 10.1097/HS9.0000000000000404 |  |  |
| 250 | EORTC 21081 | NCT01098656 | 10.1684/ejd.2017.3008 |  |  |
| 251 | EPCORE DLBCL-1 | NCT04628494 | NCT04628494 |  |  |
| 252 | EPCORE DLBCL-2 | NCT05578976 | EudraCT 2021-000168-31 |  |  |
| 253 | EPCORE FL-1 | NCT05409066 | 10.1182/blood-2022-157584 |  |  |
| 254 | EPCORE-FL2 | NCT06191744 | 10.1200/JCO.2024.42.16_suppl.TPS7084 | yes |  |
| 255 | ESCALADE,  ACE-LY-312 | NCT04529772 | EudraCT 2019-001755-39 |  |  |
| 256 | EU-MCL Elderly | NCT00209209 | 10.1056/NEJMoa1200920,  10.1200/JCO.19.01294 |  |  |
| 257 | EVAPOR | NCT04214444 | 2019-002542-20 |  |  |
| 258 | Feng 2018 | – | 10.1182/blood-2018-99-116295 |  |  |
| 259 | FIL FLAZ12 | NCT01827605 | 10.1016/j.annonc.2023.10.095, 10.1097/01.HS9.0000847444.51476.b0 |  |  |
| 260 | FIL_PREVID | NCT04442412 | 2019-004474-26 |  |  |
| 261 | FIL-DLCL04 | NCT00499018 | 10.1016/S1470-2045(17)30444-8 |  |  |
| 262 | FIL-FOLL19 | NCT05058404 | 2020-003277-22 |  |  |
| 263 | FIL-MCL0208 | NCT02354313 | 10.1016/S2352-3026(20)30358-6 |  |  |
| 264 | FIL-VERAL12 | NCT01805557 | 10.1097/01.HS9.0000843776.08811.17 |  |  |
| 265 | FIORELLA,  IELSG45 | NCT03495960 | NCT03495960 |  |  |
| 266 | First-MIND | NCT04134936 | 10.1182/blood.2023020637, 10.1200/JCO.2021.39.15_suppl.7540 |  |  |
| 267 | FLASH | NCT02448381 | 10.1001/jamadermatol.2022.2749 |  |  |
| 268 | FLINTER | NCT03976102 | NCT03976102 |  |  |
| 269 | FLIRT | NCT02303119 | 10.1200/JCO.22.02327, 10.1200/JCO.2022.40.16_suppl.7512 |  |  |
| 270 | FLYER | NCT00278421 | 10.1016/S0140-6736(19)33008-9 |  |  |
| 271 | FOLL05 | NCT00774826 | 10.1200/JCO.2017.74.1652, 10.1200/JCO.2017.74.1652 |  |  |
| 272 | FOLL12 | NCT02063685 | 10.1002/hon.3184,  10.1200/JCO.21.01234 |  |  |
| 273 | FoRT | NCT00310167 | 10.1002/hon.34_2630,  10.1016/S1470-2045(14)70036-1 |  |  |
| 274 | FORTplus | NCT05045664 | NCT05045664 |  |  |
| 275 | FRONT-MIND | NCT04824092 | 10.1200/JCO.2022.40.16_suppl.TPS7590, 10.1097/01.HS9.0000851284.83380.89 |  |  |
| 276 | G-PCNSL-SG1 | NCT00153530 | 10.1007/s00432-017-2423-5, 10.1016/S1470-2045(10)70229-1 |  |  |
| 277 | GABe2016 | NCT03492775 | NCT03492775 |  |  |
| 278 | GADOLIN | NCT01059630 | 10.1007/s00277-016-2878-5, 10.1016/S1470-2045(16)30097-3 |  |  |
| 279 | GALLIUM | NCT01332968 | 10.1056/NEJMoa1614598 |  |  |
| 280 | Gao 2018 | – | 10.1007/s00520-018-4193-2 |  |  |
| 281 | GB241NHL1 | NCT03003039 | NCT03003039 |  |  |
| 282 | GEL-R-COMP-2013 | NCT02012088 | 10.1002/cam4.3730,  10.1016/j.clml.2017.07.207 |  |  |
| 283 | GITMO/IIL | NCT00435955 | 10.1182/blood-2007-10-116749, 10.3324/haematol.2018.209932 |  |  |
| 284 | GLOBRYTE | NCT06084936 | 10.1016/S2152-2650(24)01599-4,  10.1182/blood-2023-173946 | yes |  |
| 285 | GLORIA | NCT05018520 | NCT05018520 |  |  |
| 286 | GLSG-OSHO70 | 2005-005473-29 | 10.1097/HS9.0000000000000600 |  |  |
| 287 | GO29365 | NCT02257567 | 10.3324/haematol.2023.283557, 10.1200/JCO.19.00172 |  |  |
| 288 | GO40150 | NCT03369964 | NCT03369964 |  |  |
| 289 | GO40515 | NCT03677141 | 10.1182/blood-2020-136295 |  |  |
| 290 | GO40516 | NCT03671018 | EudraCT 2018-001141-13 |  |  |
| 291 | GOAL 2 | 2019-002373-59 | EudraCT 2019-002373-59 |  |  |
| 292 | GOELAMS 02-03 | NCT00841945 | 10.1182/blood-2017-07-793984 |  |  |
| 293 | GOELAMS 075 | NCT00561379 | 10.1016/j.bbmt.2009.12.530 |  |  |
| 294 | Gorenkova 2020 | – | 10.1016/S2152-2650(20)30882-X |  |  |
| 295 | GOSPEL I | ACTRN12620000594921 | 10.1186/s13063-020-04945-4 |  |  |
| 296 | GOYA | NCT01287741 | 10.1186/s41687-024-00708-w, 10.1002/cam4.4692,  10.1186/s13045-020-00900-7 |  |  |
| 297 | Guidance-01 | NCT04025593 | 10.1002/hon.2879,  10.1016/j.ccell.2023.09.004 |  |  |
| 298 | Hafez 2018 | – | 10.1016/j.ajme.2018.07.006,  10.1016/S2152-2650(22)01486-0 |  |  |
| 299 | He 2020 | – | https://e-century.us/files/ijcem/13/10/  ijcem0115835.pdf |  |  |
| 300 | HELYX | NCT00154440 | 10.1007/s00535-018-1517-4 |  |  |
| 301 | HERILY | CTRI/2013/08/003921 | 10.4103/ijmpo.ijmpo_25_17, https://japi.org/article/files/Efficacy__Safety_and_Immunogenecitystudy_of_intravenous_infusion_of_rituximab__hetero__and_reference_medicinal_product__rituximab_Roche__in_indian_patients_of_follicular_lymphoma_preliminary_report__herily_.pdf.pdf |  |  |
| 302 | Hi-CHOP | NCT03485118 | ChiCTR2100042104 |  |  |
| 303 | Hirode 2024 | – | 10.1097/HEP.0000000000001077 | yes |  |
| 304 | HLX01-FL03 | NCT04671420 | NCT04671420 |  |  |
| 305 | HLX01-NHL02 | NCT02584920 | 10.21147/j.issn.1000-9604.2021.03.11 |  |  |
| 306 | HLX01-NHL03 | NCT02787239 | 10.1182/blood-2019-130603,  10.1186/s13045-020-00871-9 |  |  |
| 307 | HOMER | NCT01200589 | 10.1182/bloodadvances.2020001942 |  |  |
| 308 | HOVON 75 MCL | NTR1772 | 10.1111/bjh.16567 |  |  |
| 309 | HOVON-84 | 2006-005174-42 | 10.1016/j.ejca.2024.115144, 10.1200/JCO.19.03418, 10.2967/jnumed.121.262205 |  |  |
| 310 | HOVON 105,  ALLG NHL 24 | 2009-014722-42, ACTRN12610000908033 | 10.1093/neuonc/noad224,  10.1016/S1470-2045(18)30747-2,  10.1093/neuonc/noab021 |  |  |
| 311 | HOVON 110,  REBEL | NTR3028 | 10.1182/blood-2022-156085 | yes |  |
| 312 | HOVON 127,  SAKK 37/16 | 2013-004394-27 | 10.1016/S2352-3026(23)00279-X, https://library.ehaweb.org/eha/2022/eha2022-congress/366213/med.chamuleau.r-codox-m.r-ivac.versus.dose-adjusted28da29-epoch-r.in.patients.html?f=menu%3D6%2Abrowseby%3D8%2Asortby%3D2%2Amedia%3D3%2Ace_id%3D2233%2Amarker%3D1750%2Afeatured%3D17676 |  |  |
| 313 | HOVON 170 | NCT06220032 | 10.1186/s40959-025-00303-y | yes |  |
| 314 | Hu 2017 | – | 10.1097/MD.0000000000008494 |  |  |
| 315 | Huang 2018 | – | 10.1200/JCO.2018.36.15_suppl.7560 |  |  |
| 316 | Huang 2024 | – | 10.3760/cma.j.cn431274-20230912-00252 | yes |  |
| 317 | ICP-CL-00113 | NCT05051891 | NCT05051891 |  |  |
| 318 | IELSG-19 | NCT00210353 | 10.1200/JCO.2016.70.6994, 10.1200/JCO.2011.40.6272 |  |  |
| 319 | IELSG32 | NCT01011920 | 10.1016/S2352-3026(17)30174-6 |  |  |
| 320 | IELSG37 | NCT01599559 | 10.1200/JCO-24-01373 | yes |  |
| 321 | IELSG43,  MATRix | NCT02531841 | 10.1182/blood-2022-171733 |  |  |
| 322 | ILyAD | NCT03078855 | 10.1016/j.eclinm.2024.102959,  10.1182/blood-2019-127320, 10.1200/JCO.2018.36.15_suppl.TPS7587 |  |  |
| 323 | IN-MIND | NCT04680052 | 10.1182/blood-2024-212970 |  |  |
| 324 | INCA | NCT01679119 | 10.1097/HS9.0000000000000404 |  |  |
| 325 | INCB 39110-211 | NCT04071366 | 10.1182/blood-2023-180205, https://ebmt2022.abstractserver.com/program/#/details/presentations/1149 |  |  |
| 326 | iNNOVATE | NCT02165397 | 10.1056/NEJMoa1802917,  10.1200/JCO.21.00838 |  |  |
| 327 | IVIG-DLBCL | KCT0005626 | KCT0005626 |  |  |
| 328 | JASMINE | NCT02747043 | 10.1007/s11523-020-00748-4, CTRI/2017/12/010935 |  |  |
| 329 | JAVELIN DLBCL | NCT02951156 | 10.1007/s11523-021-00849-8 |  |  |
| 330 | JCOG0203 | NCT00147121 | 10.1200/JCO.2011.34.8508,  10.1016/S2352-3026(18)30155-8 |  |  |
| 331 | JCOG0601 | jRCTs031180139 | 10.1182/bloodadvances.2020002567 |  |  |
| 332 | JCOG0908 | jRCTs031180103 | 10.1111/cas.14604 |  |  |
| 333 | JCOG1111C | jRCTs031180169 | jRCTs031180169 |  |  |
| 334 | JCOG1114C | jRCTs031180207 | 10.1093/neuonc/noac246 |  |  |
| 335 | JCOG1411,  FLORA | UMIN000025187 | 10.1093/jjco/hyy085 |  |  |
| 336 | JCOG2008,  MAIN | jRCT1031210379 | jRCT1031210379 |  |  |
| 337 | JCOG2219,  TRANSFER | jRCTs031240169 | 10.1093/jjco/hyae144 | yes |  |
| 338 | JCOG9801 | NCT00145002 | 10.1200/JCO.2007.11.9958,  10.1182/blood-2018-99-112734 |  |  |
| 339 | KILT | NCT04984837 | NCT04984837 |  |  |
| 340 | Knudsen 2023 | – | 10.1016/j.clnesp.2023.09.566 | yes |  |
| 341 | LEGEND | NCT02060656 | 10.1007/s00277-019-03842-4 |  |  |
| 342 | LIFE-L | NCT05839210 | 10.1158/1538-7445.AM2023-3218 | yes |  |
| 343 | Liu 2019 | – | https://e-century.us/files/ijcem/12/9/ ijcem0097217.pdf |  |  |
| 344 | Liu 2021 | – | https://www.ncbi.nlm.nih.gov/pmc/articles/PMC8205818/pdf/ajtr0013-5362.pdf |  |  |
| 345 | Liu 2023 | – | 10.1182/blood-2023-189933 | yes |  |
| 346 | LIVE | NTR5953 | 10.2196/27886,  10.1186/s13063-017-1943-2 |  |  |
| 347 | LNH03-6B | NCT00144755 | 10.1016/S1470-2045(13)70122-0 |  |  |
| 348 | LOC-R01 | NCT04446962 | 10.1186/s13045-024-01606-w |  |  |
| 349 | LOTIS-5 | NCT04384484 | NCT04384484 |  |  |
| 350 | LOTIS-6 | NCT04699461 | 10.1002/hon.176_2880 |  |  |
| 351 | LUMIERE | NCT01482962 | 10.1200/JCO.18.00899 |  |  |
| 352 | Luo 2023 | – | https://e-century.us/files/ijcem/16/ 4/ijcem0143391.pdf | yes |  |
| 353 | Lv 2023 | – | 10.1182/blood-2023-182681 | yes |  |
| 354 | LY.12 | NCT00078949 | 10.1200/JCO.2013.53.9593 |  |  |
| 355 | LY.17 | NCT02436707 | 10.1111/bjh.19555 | yes |  |
| 356 | LYM-3002 | NCT00722137 | 10.1016/S1470-2045(18)30685-5 |  |  |
| 357 | LYM1 | NCT00005589 | 10.1038/s41409-020-01182-w, 10.1200/JCO.2012.47.1862 |  |  |
| 358 | LyMa | NCT00921414 | 10.1200/JCO.23.01586,  10.1056/NEJMoa1701769 |  |  |
| 359 | Lymfit (pilot study) | NCT05259657 | 10.3390/healthcare12111101 | yes |  |
| 360 | Lymfit_RCT | NCT05257785 | NCT05257785 |  |  |
| 361 | Lymphoseed | NCT03260231 | NCT03260231 |  |  |
| 362 | LympSCare | NCT05443165 | NCT05443165 |  |  |
| 363 | LYMRIT-37-01 | NCT01796171 | 10.1182/blood-2018-99-110555 |  |  |
| 364 | LYMRIT-37-02 | NCT02657447 | NCT02657447 |  |  |
| 365 | M-PUVA-2012 | NCT01686594 | 10.3389/fmed.2020.00330,  10.1001/jamadermatol.2018.5905 |  |  |
| 366 | Ma 2024 | – | https://cstj.cqvip.com/Qikan/Article/Detail?id=7111898616 | yes |  |
| 367 | MA-ASCT-II-001 | NCT05156554 | NCT05156554 |  |  |
| 368 | MABCUTE | NCT01461928 | 10.3324/haematol.2020.274803, 10.1002/hon.43_2630 |  |  |
| 369 | MabEase | NCT01649856 | 10.3324/haematol.2017.173583 |  |  |
| 370 | MAGNIFY | NCT01996865 | 10.1200/JCO.2020.38.15_suppl.8046, 10.1182/blood-2021-145640,  10.1159/000502425 |  |  |
| 371 | MAHOGANY | NCT05100862 | NCT05100862 |  |  |
| 372 | MANGROVE | NCT04002297 | NCT04002297 |  |  |
| 373 | Marchesi 2018 | – | 10.1111/trf.14533,  10.1038/s41409-018-0354-7 |  |  |
| 374 | MARSUN | NCT06006117 | 10.1182/blood-2023-180473 | yes |  |
| 375 | MAVORIC | NCT01728805 | 10.1016/S1470-2045(18)30379-6 |  |  |
| 376 | MCL Younger | NCT00209222 | 10.1111/bjh.19854,  10.1016/S0140-6736(16)00739-X |  |  |
| 377 | MCL-R2 Elderly | NCT01865110 | 10.1182/blood-2023-188788,  10.1002/hon.2440 |  |  |
| 378 | Mecapegfilgrastim-Lym-2020-1 | NCT04460508 | NCT04460508 |  |  |
| 379 | MIDAS | NCT03380026 | 10.1007/s13555-022-00681-6 |  |  |
| 380 | MIL62-CT301 | NCT04834024 | NCT04834024 |  |  |
| 381 | ML29496 | NCT02498951 | 10.1182/blood-2020-134457 |  |  |
| 382 | Mou 2017 | – | 10.7534/j.issn.1009-2137.2017.05.021 |  |  |
| 383 | Muhebaier 2018 | – | https://caod.oriprobe.com/articles/55624611/Efficacy_Prognosis_and_Safety_of_Rituximab_Combine.htm |  |  |
| 384 | Munjal 2023 | – | 10.1016/j.jid.2023.03.700 | yes |  |
| 385 | Nastoupil 2017 | – | 10.1111/bjh.14541 |  |  |
| 386 | NAVAL-1 | NCT05011058 | 10.1016/j.htct.2024.09.431 | yes |  |
| 387 | NEUPERSART | NCT03805867 | NCT03805867 |  |  |
| 388 | NFL2016-B1 | NCT03018626 | NCT03018626 |  |  |
| 389 | NFL2016-B2 | NCT03016000 | NCT03016000 |  |  |
| 390 | NHL-001 | NCT01852435 | 10.1016/S2352-3026(19)30051-1 |  |  |
| 391 | NHL-014,  PEARL | NCT05179733 | NCT05179733 |  |  |
| 392 | NHL-PD1-1 | NCT04789434 | NCT04789434 |  |  |
| 393 | NHL-PD1-2 | NCT04799314 | NCT04799314 |  |  |
| 394 | NIVEAU | NCT03366272 | 10.1159/000540557, 10.1182/blood-2020-136941,  10.1182/blood-2020-139459 |  |  |
| 395 | NKT-SYSUCC-2013 | NCT02085655 | 10.1002/hon.119_2629,  10.1002/hon.2437_114 |  |  |
| 396 | NLG-LBC7,  POLAR BEAR | NCT04332822 | 10.1097/01.HS9.0000967820.91359.ec |  |  |
| 397 | NTP-H02-I-III | NCT05040906 | NCT05040906 |  |  |
| 398 | OASIS-2 | NCT04802590 | NCT04802590 |  |  |
| 399 | OLYMPIA-1 | NCT06091254 | 10.1200/JCO.2024.42.16_suppl.TPS7096 | yes |  |
| 400 | OLYMPIA-2 | NCT06097364 | 10.1200/JCO.2024.42.16_suppl.TPS7099 | yes |  |
| 401 | OLYMPIA-3 | NCT06091865 | 10.1200/JCO.2024.42.16_suppl.TPS7086 | yes |  |
| 402 | OLYMPIA-4 | NCT06230224 | 10.1200/JCO.2024.42.16_suppl.TPS7093 | yes |  |
| 403 | OLYMPIA-5 | NCT06149286 | 10.1200/JCO.2024.42.16_suppl.TPS7094 | yes |  |
| 404 | OPTIMAL >60 | NCT01478542 | 10.1002/hon.2437_119, 10.1182/blood.V130.Suppl_1.1549.1549 |  |  |
| 405 | OPTIMATE | NCT04931368, DRKS00022768 | NCT04931368 |  |  |
| 406 | ORACLE | NCT03703375 (Japan), NCT03593018 (Europe) | 10.1016/S2352-3026(24)00102-9 |  |  |
| 407 | ORCHARRD | NCT01014208 | 10.1200/JCO.2016.69.0198 |  |  |
| 408 | P+R-ICE | NCT05221645 | NCT05221645 |  |  |
| 409 | PALS | NCT05595577 | NCT05595577 |  |  |
| 410 | PALS | NCT01719562 | 10.1186/s40814-024-01580-7 | yes |  |
| 411 | PCNSL-001 | NCT05334238 | NCT05334238 |  |  |
| 412 | Peng 2020 | – | 10.1080/10428194.2020.1747061 |  |  |
| 413 | Perales 2022 | NCT05664217 | 10.1182/blood-2022-162511 | yes |  |
| 414 | PERSPECTIVE | NCT02947347 | 10.1002/ajh.26005,  10.1111/bjh.16638,  10.1158/1538-7445.AM2020-CT290 |  |  |
| 415 | PETAL | NCT00554164 | 10.1200/JCO.2017.76.8093 |  |  |
| 416 | PETReA | 2016-004010-10 | ISRCTN86739591 |  |  |
| 417 | PHARAOM | NCT04670029 | 10.2196/40969 |  |  |
| 418 | PHII-203 | NCT04541017 | NCT04541017 |  |  |
| 419 | PHOENIX | NCT01855750 | 10.1182/bloodadvances.2022009389, 10.1200/JCO.18.02403 |  |  |
| 420 | PICASSO,  CISL-1703 | NCT03123718 | 10.1097/01.HS9.0000967828.60026.bd |  |  |
| 421 | PILLAR2 | NCT00790036 | 10.1093/annonc/mdx764 |  |  |
| 422 | PIVOTAL 201 | NCT00168064 | 10.1016/j.clml.2023.08.020, 10.1001/2013.jamadermatol.541 |  |  |
| 423 | PIX301 | NCT00088530 | 10.1016/S1470-2045(12)70212-7,  10.1007/s40261-018-0635-3 |  |  |
| 424 | PIX306 | NCT01321541 | 10.1111/bjh.16255,  10.2217/fon-2016-0137 |  |  |
| 425 | PLM60-MC-1201 | NCT03553914 | NCT03553914 |  |  |
| 426 | PLM60-PK | NCT05173545 | NCT05173545 |  |  |
| 427 | PLRG4 | NCT00801281 | 10.1111/bjh.16264 |  |  |
| 428 | PMBL-2022 | NCT06188676 | 10.1016/S2152-2650(24)01538-6 | yes |  |
| 429 | Pola-R-ICE | NCT04833114 | 10.1159/000533576 |  |  |
| 430 | POLARGO | NCT04182204 | 10.1200/JCO.2022.40.16_suppl.7551 |  |  |
| 431 | POLARIX | NCT03274492 | 10.1182/blood-2022-157761, 10.1056/NEJMoa2115304 |  |  |
| 432 | PRECIS | NCT00863460 | 10.1200/JCO.18.00306 |  |  |
| 433 | PrefMab | NCT01724021 | 10.1093/annonc/mdw685 |  |  |
| 434 | PRIMA | NCT00140582 | 10.1016/S0140-6736(10)62175-7, 10.1179/1607845414Y.0000000179, 10.1200/JCO.19.01073 |  |  |
| 435 | PRIMA-CNS | NCT06830421 | 10.1186/s12885-023-11193-7 | yes |  |
| 436 | PRIMO | NCT03372057 | 10.1182/blood-2019-121401 |  |  |
| 437 | PROTECT | NCT06449625 | 10.1136/bmjopen-2024-089862 | yes |  |
| 438 | PTCL-001 | NCT04747236 | NCT04747236 |  |  |
| 439 | PUMCH-NHL-011 | NCT05054426 | NCT05054426 |  |  |
| 440 | Qian 2021 | – | https://e-century.us/files/ajtr/13/8/  ajtr0132413.pdf |  |  |
| 441 | QUILT-3.092 | NCT05618925 | NCT05618925 |  |  |
| 442 | R-CHOP 14 vs 21 | ISCRTN16017947 | 10.1016/S0140-6736(13)60313-X, 10.1093/annonc/mdx128 |  |  |
| 443 | R-HAD | NCT01449344 | 10.1038/s41375-024-02254-2 | yes |  |
| 444 | R-MegaCHOEP | NCT00129090 | 10.1016/S1470-2045(12)70481-3, 10.1016/S2352-3026(21)00022-3 |  |  |
| 445 | RABBIT-14 | UMIN000008702 | 10.1080/10428194.2017.1390233, 10.1182/blood.V128.22.5325.5325 |  |  |
| 446 | RADL | CTRI/2017/10/010152 | CTRI/2017/10/010152 |  |  |
| 447 | RAINBOW | NCT04061512 | NCT04061512 |  |  |
| 448 | RAMO-2 | NCT02809053 | CTRI/2017/07/009035 |  |  |
| 449 | RAY | NCT01646021 | 10.1080/10428194.2017.1326034,  10.1016/S0140-6736(15)00667-4, 10.1182/blood-2015-03-635326, 10.1038/s41375-018-0023-2 |  |  |
| 450 | REFLECTIONS | NCT02213263 | 10.1007/s40259-019-00398-7 |  |  |
| 451 | REFRACT | NCT05848765 | 10.1186/s12885-024-12112-0 | yes |  |
| 452 | REFUEL | NCT05130099 | 10.2196/preprints.69336 |  |  |
| 453 | REIL | NCT02272751 | 10.1007/s11764-020-00941-4,  10.1186/s13102-019-0127-7 |  |  |
| 454 | RELEVANCE | NCT01650701 | 10.1200/JCO.22.00843,  10.1056/NEJMoa1805104 |  |  |
| 455 | RELIANCE | NCT04089215 | 10.1002/cam4.3686, 10.1200/JCO.2022.40.16_suppl.7529, 10.1182/blood-2021-148358 |  |  |
| 456 | Relyage | NCT04113226 | NCT04113226 |  |  |
| 457 | REMARC | NCT01122472 | 10.1200/JCO.2017.72.6984 |  |  |
| 458 | REMoDL-A | NCT04546620 | NCT04546620 |  |  |
| 459 | REMoDL-B | NCT01324596 | 10.1182/blood-2022-159442,  10.1016/S1470-2045(18)30935-5 |  |  |
| 460 | RENOIR | NCT02390869 | 10.1182/blood-2023-174901 | yes |  |
| 461 | RESILIENCE | NCT05223413 | NCT05223413 |  |  |
| 462 | RESMAIN | NCT02953301 | 10.2139/ssrn.4862663,  10.1016/S0959-8049(21)00724-3 |  |  |
| 463 | RICOVER-60 | NCT00052936 | 10.1016/S1470-2045(08)70002-0 |  |  |
| 464 | RITU_CYTO | KCT0005708 | KCT0005708 |  |  |
| 465 | RIVA | NCT03307746,  2017–000302-37 | 0.1186/s13063-018-2996-6 |  |  |
| 466 | RJ-NHL-1805 | NCT03600363 | NCT03600363 |  |  |
| 467 | Ro-CHOP | NCT01796002 | 10.1200/JCO.23.01687,  10.1200/JCO.21.01815 |  |  |
| 468 | ROMULUS | NCT01691898 | 10.1016/S2352-3026(19)30026-2 |  |  |
| 469 | ROSE,  CISL-1504 | NCT02445404 | 10.3389/fonc.2023.1230629, 10.1002/hon.146_2631 |  |  |
| 470 | ROSEWOOD | NCT03332017 | 10.1080/03007995.2024.2409837, 10.1097/01.HS9.0000843712.33707.fe |  |  |
| 471 | RTOG-1114 | NCT01399372 | 10.1200/JCO.2020.38.15_suppl.2501 |  |  |
| 472 | RTXM83-AC-01-11 | NCT02268045 | 10.1080/10428194.2019.1633632 |  |  |
| 473 | SABRINA | NCT01200758 | 10.1016/S2352-3026(17)30078-9 |  |  |
| 474 | SAKK 35/03 | NCT00227695 | 10.1182/bloodadvances.2020002858, 10.1182/blood-2018-99-116864 |  |  |
| 475 | SAKK 35/10 | NCT01307605 | 10.1182/bloodadvances.2024014840, 10.1182/blood-2018-10-879643 |  |  |
| 476 | SAKK 35/14 | NCT02451111 | 10.1002/hon.3163_80 |  |  |
| 477 | SCT400NHL3 | NCT02772822 | 10.1002/hon.3054,  10.1182/blood-2021-149861 |  |  |
| 478 | SCUBA-1 | CTRI/2019/07/020236 | 10.1007/s12288-022-01562-w7 |  |  |
| 479 | SELENE | NCT01974440 | 10.1182/bloodadvances.2023010298 | yes |  |
| 480 | SENIOR | NCT02128061 | 10.1200/JCO.20.02666,  10.1182/blood-2019-123612 |  |  |
| 481 | SENTINEL | NCT03154710 | 10.2196/preprints.65960 | yes |  |
| 482 | SEXIE-R-CHOP | NCT00290667 | 10.1111/bjh.14860 |  |  |
| 483 | SHIELD | – | 10.1182/blood-2021-153987 |  |  |
| 484 | SHINE | NCT01776840 | 10.1159/000526456,  10.1056/NEJMoa2201817 |  |  |
| 485 | SIBP-02-03 | NCT04361279 | NCT04361279 |  |  |
| 486 | SIESTA | 2015-005688-18 | 10.1182/bloodadvances.2021006330, 10.1182/blood-2021-144659 |  |  |
| 487 | SOLAR | NCT03713320 | NCT03713320 |  |  |
| 488 | Song 2021 | – | 10.1182/blood-2021-150085 |  |  |
| 489 | Song 2017 | – | 10.1080/10428194.2016.1213838 |  |  |
| 490 | SPRINT,  MCL-002 | NCT00875667 | 10.1016/S1470-2045(15)00559-8 |  |  |
| 491 | STARGLO,  GO41944 | NCT04408638 | 10.1016/S0140-6736(24)01774-4 |  |  |
| 492 | STAY-STRONG | NCT05556239 | NCT05556239 | yes |  |
| 493 | STELLAR | NCT03899337 | 10.1186/s12885-019-5717-y |  |  |
| 494 | Stepanishyna 2020 | – | 10.1182/blood-2020-141348, 10.1097/HS9.0000000000000566, 10.1097/HS9.0000000000000404 |  |  |
| 495 | StiL NHL1 | NCT00991211 | 10.1016/S0140-6736(12)61763-2, 10.1200/JCO.2017.35.15_suppl.7501 |  |  |
| 496 | StiL NHL7,  MAINTAIN | NCT00877214 | 10.1159/000492737,  10.1182/blood-2019-121909 |  |  |
| 497 | STOP-CA | NCT02943590 | 10.1001/jama.2023.11887, 10.1080/10428194.2024.2317343 | yes |  |
| 498 | SUNMO | NCT05171647 | NCT05171647 |  |  |
| 499 | SWOG 9704 | NCT00004031 | 10.1056/NEJMoa1301077 |  |  |
| 500 | SWOG S0016 | NCT00006721 | 10.1200/JCO.2012.42.4101, 10.1200/JCO.2017.74.5083 |  |  |
| 501 | SWOG S1106 | NCT01412879 | 10.1111/bjh.14480 |  |  |
| 502 | SWOG S1608 | NCT03269669 | 10.1182/blood-2021-146182 |  |  |
| 503 | SWOG S1918 | NCT04799275 | 10.1016/j.jgo.2021.10.003 |  |  |
| 504 | SWOG S2005 | NCT04840602 | NCT04840602 |  |  |
| 505 | SWOG S2114 | NCT05633615 | NCT05633615 |  |  |
| 506 | SYMPATICO | NCT03112174 | 10.1016/S1470-2045(24)00682-X, 10.1186/s13045-021-01188-x |  |  |
| 507 | SYMPHONY-1,  EZH-302 | NCT04224493 | [NCT04224493](https://www.german-lymphoma-alliance.de/media/public/E700B9F5-8FC1-9982-DBD4-078456A141A6/EZH-302-Synopsis-05Nov19.pdf?ts=1576675339) |  |  |
| 508 | Tao 2020 | – | 10.1111/bcpt.13494 |  |  |
| 509 | tele@home | NCT05779605 | 10.1186/s40959-024-00249-7 | yes |  |
| 510 | TEMPO | NCT04038359 | 10.1097/01.HS9.0000847360.49139.75 |  |  |
| 511 | TenoCore | NCT02585947 | 10.3346/jkms.2023.38.e216,  10.1007/s12072-022-10337-4 |  |  |
| 512 | THEORY | NCT05675813 | NCT05675813 |  |  |
| 513 | TIDAL | NCT03768505 | 10.1182/blood-2022-165409, 10.1200/JCO.2022.40.16_suppl.7511 |  |  |
| 514 | TOP-FLOR | NCT05788081 | 10.1200/JCO.2024.42.16_suppl.TPS7092, 10.1182/blood-2023-173110 | yes |  |
| 515 | TOTEM-01 | NCT05205902 | NCT05205902 |  |  |
| 516 | TRANSCRIPT | NCT05444712 | NCT05444712 |  |  |
| 517 | TRANSFORM | NCT03575351 | 10.1182/blood-2021-151611 |  |  |
| 518 | TRAVERSE | NCT05951959 | 10.1182/blood-2023-174718 | yes |  |
| 519 | TRIANGLE | NCT02858258 | 10.1016/S0140-6736(24)00184-3,  10.1182/blood-2022-163018 |  |  |
| 520 | TROG 99.03,  ALLG NHLLOW5 | NCT00115700 | 10.1200/JCO.2018.77.9892, 10.1101/2024.08.09.24311704 |  |  |
| 521 | UNFOLDER | NCT00278408 | 10.1097/HS9.0000000000000904, 10.1200/JCO.2018.36.15_suppl.7574 |  |  |
| 522 | UNITY-NHL | NCT02793583 | 10.1200/JCO.20.03433,  10.1182/blood-2021-147425 |  |  |
| 523 | VEGA | NCT05564052 | NCT05564052 |  |  |
| 524 | VITALIZE | NCT04920617 | NCT04920617 |  |  |
| 525 | VIWA-1 | NCT05099471 | NCT05099471 |  |  |
| 526 | VT-EBV-201 | NCT03671850 | NCT03671850 |  |  |
| 527 | Wang 2023 | – | 10.3760/cma.j.cn115356-20211125-00273 | yes |  |
| 528 | Watch and Wait | NCT00112931 | 10.1111/bjh.19918,  10.1016/S1470-2045(14)70027-0 |  |  |
| 529 | WAVELINE-003 | NCT05139017 | 10.1200/JCO.2022.40.16_suppl.TPS7592 |  |  |
| 530 | WAVELINE-006 | NCT05458297 | 10.1200/JCO.2023.41.16_suppl.TPS7595 |  |  |
| 531 | Wei 2020 | – | 10.20892/j.issn.2095-3941.2020.0160 |  |  |
| 532 | XmAb13676-03 | NCT05328102 | 10.1097/01.HS9.0000851240.74602.7c |  |  |
| 533 | XPORT-DLBCL-030 | NCT04442022 | 10.1182/blood-2021-150025 |  |  |
| 534 | Yang 2018 | – | 10.3389/fphar.2018.00991 |  |  |
| 535 | YO42207 | NCT04660799 | 10.1080/10428194.2024.2439525, 10.1097/01.HS9.0000975952.54634.fd |  |  |
| 536 | ZAR2007 | NCT00662948 | 10.1080/10428194.2021.1971216, 10.1182/blood.V122.21.369.369 |  |  |
| 537 | ZEBRA | NCT05635162 | NCT05635162 |  |  |
| 538 | Zeng 2017 | – | 10.3969/j.issn.2095-1264.2017.04.22, http://61.187.87.56:81/article/  detail.aspx?id=672932280 |  |  |
| 539 | Zhang 2020 | – | https://www.jbuon.com/archive/25-2-1042.pdf |  |  |
| 540 | Zhang 2021 | – | 10.3760/cma.j.issn.1009-9921.2018.01.010 |  |  |
| 541 | Zhang 2023 (a) | – | 10.3892/ol.2023.14027 | yes |  |
| 542 | Zhang 2023 (b) | – | 10.1182/blood-2023-188245 | yes |  |
| 543 | Zhen 2019 | – | 10.1182/blood-2019-128438 |  |  |
| 544 | Zheng 2020 | – | 10.1016/j.annonc.2020.08.027 |  |  |
| 545 | ZILO-301 | NCT05431179 | 10.1097/01.HS9.0000847484.83317.ef |  |  |
| 546 | Zoellner 2021 | – | 10.1016/S2352-3026(21)00195-2,  10.1182/blood-2004-10-3883 |  |  |
| 547 | ZUMA-7 | NCT03391466 | 10.1182/blood.2022015478, 10.1056/NEJMoa2116133 |  |  |
| 548 | ZUMA-22 | NCT05371093 | NCT05371093 |  |  |
| 549 | ZUMA-23 | NCT05605899 | 10.1200/JCO.2023.41.16_suppl.TPS7578 |  |  |

# Appendix 4. Use of patient-reported outcome measures (PROM) in included trials

| **Name of PROM** | **Scope of application** | **Frequency** | **Trials referencing PROM** (registration number or first author/year) |
| --- | --- | --- | --- |
| EORTC QLQ-Core questionnaire (EORTC QLQ-C30) | Condition-specific (cancer) | 75 | NTR5953, NCT06830421, NCT06449625, NCT06230224 NCT06220032, NCT06191744 NCT06149286, NCT06097364, NCT06091865, NCT06091254, NCT06084936, NCT05788081, NCT05605899, NCT05556239, NCT05376709, NCT05371093, NCT05298293, NCT05205902, NCT05179733, NCT05171647, NCT05130099, NCT05100862, NCT05045664, NCT05018520, NCT04931368, NCT04712097, NCT04670029, NCT04546620, NCT04442412, NCT04408638, NCT04384484, NCT04182204, NCT04002297, NCT03703375 (Japan) and NCT03593018 (Europe), NCT03575351, NCT03492775, NCT03391466, NCT03332017, NCT03274492, NCT03154710, NCT03053440, NCT02972840, NCT02763319, NCT02531841, NCT02498951, NCT02390869, NCT02354313, NCT02313389, NCT02278796, NCT02272751, NCT02128061, NCT02044276, NCT01870479, NCT01827605, NCT01796002, NCT01679119, NCT01650701, NCT01399372, NCT01324596, NCT01287741, NCT01197560, NCT00877006, NCT00875667, NCT00722137, NCT00662948, NCT00153530, NCT00140582, NCT00014326, jRCTs051180026, ISRCTN11038174, 2019-002373-59, 2009-014722-42, Chuang 2017, He 2020, Knudsen 2023 |
| FACT-Lymphoma (FACT-Lym) | Condition-specific (NHL) | 54 | NCT06230224, NCT06191744, NCT06149286, NCT06097364, NCT06091865, NCT06091254, NCT05848765, NCT05788081, NCT05595577, NCT05179733, NCT05171647, NCT05099471, NCT05058404, NCT05045664, NCT04920617, NCT04712097, NCT04699461, NCT04628494, NCT04546620, NCT04442412, NCT04408638, NCT04384484, NCT04263480, NCT04224493, NCT04182204, NCT03575351, NCT03570892, NCT03505762, NCT03274492, NCT03112174, NCT02972840, NCT02964858, NCT02951156, NCT02272751, NCT01996865, NCT01974440, NCT01855750, NCT01776840, NCT01719562, NCT01646021, NCT01482962, NCT01415752, NCT01332968, NCT01287741, NCT01232556, NCT01216683, NCT01077518, NCT01059630, NCT01014208, NCT00078949, ACTRN12620000594921, ACTRN12617000068369, Gao 2018, Luo 2023 |
| EuroQol 5 Dimensions (EQ-5D) | Generic | 51 | NCT06230224, NCT06191744, NCT06149286, NCT06097364, NCT06091865, NCT06091254, NCT06006117, NCT05848765, NCT05605899, NCT05444712, NCT05371093, NCT05223413, NCT05171647, NCT05130099, NCT05100862, NCT04920617, NCT04699461, NCT04384484, NCT04224493, NCT04061512, NCT04002297, NCT03703375 (Japan) and NCT03593018 (Europe), NCT03570892, NCT03391466, NCT03366272, NCT03332017, NCT03274492, NCT03112174, NCT03053440, NCT02972840, NCT02951156, NCT02763319, NCT02390869, NCT02165397, NCT01974440, NCT01855750, NCT01776840, NCT01728805, NCT01650701, NCT01646021, NCT01578499, NCT01332968, NCT01232556, NCT01197560, NCT01077518, NCT01059630, NCT01014208, NCT00722137, NCT00310167, ACTRN12617000068369, 2016-004010-10 |
| FACT-General (FACT-G) | Condition-specific (cancer) | 18 | NCT06149286, NCT05443165, NCT04662255, NCT02964858, NCT02953301, NCT01728805, NCT01578499, NCT01332968, NCT01216683, NCT01059630, NCT01014208, NCT00662948, NCT00140582, NCT00112931, NCT00075946,  2016-004010-10, Hafez 2018, Munjal 2023 |
| Hospital Anxiety and Depression Scale (HADS) | Symptom-specific | 11 | NTR5953, NCT05556239, NCT04701554, NCT02272751, NCT01870479, NCT01686594, NCT00112931, NCT00075946, ACTRN12617000068369, 2016-004010-10, Gao 2018 |
| EORTC QLQ-Brain Cancer (EORTC QLQ-BN20) | Condition-specific (brain cancer) | 8 | NCT06830421, NCT04931368, NCT02531841, NCT02498951, NCT02313389, NCT01399372, NCT00153530, 2009-014722-42 |
| Pruritus visual analogue scale (VAS) | Symptom-specific | 8 | NCT03454945, NCT03380026, NCT03292406, NCT03011814, NCT02953301, NCT02811783, NCT02213861, NCT01625455 |
| Skindex-29 | Symptom-specific | 7 | NCT05205902, NCT03292406, NCT02953301, NCT02213861, NCT01728805, NCT01578499, Munjal 2023 |
| FACT/GOG-Neurotoxicity (FACT/GOG-Ntx) | Side effect-specific | 6 | NCT05171647, NCT04182204, NCT03505762, NCT03274492, NCT01415752, NCT01216683 |
| Patient-Reported Outcomes version of the Common Terminology Criteria for Adverse Events (PRO-CTCAE) | Side effect-specific | 6 | NCT06220032, NCT06191744, NCT04803201, NCT04699461, NCT04665115, NCT04662255 |
| Short-Form-36 (SF-36) | Generic | 6 | UMIN000026758, NCT05779605, NCT05130099, NCT03570892, NCT02983942, NCT01719562 |
| EORTC QLQ-NHL-High grade  (EORTC QLQ-NHL-HG29) | Condition-specific  (high grade NHL) | 5 | NTR5953, NCT06220032, NCT05605899, NCT03899337, 2019-002373-59 |
| Patient Global Impression of Change (PGIC) | PRE | 5 | NCT06191744, NCT06149286, NCT06097364, NCT06091865, NCT06091254 |
| Patient Global Impression of Severity (PGIS) | PRE | 5 | NCT06191744, NCT06149286, NCT06097364, NCT06091865, NCT06091254 |
| Dermatology Life Quality Index (DLQI) | Symptom-specific | 4 | NCT03454945, NCT02323659, NCT01686594, NCT01625455 |
| Brief Fatigue Inventory (BFI) | Symptom-specific | 3 | NCT02951156, NCT00722137, Chuang 2017 |
| FACIT-Fatigue (FACIT-F) | Symptom-specific | 3 | NCT05595577, NCT01415752, NCT01216683 |
| MD Anderson Symptom Inventory (MDASI) | Symptom-specific | 3 | NCT05556239, NCT05181540, NCT01691898 |
| Mental Adjustment to Cancer (MAC) Scale | PRE | 3 | NTR5953, NCT00112931, NCT00075946 |
| Multidimensional Fatigue Inventory (MFI) | Symptom-specific | 3 | NTR5953, NCT04670029, IRCT20130616013690N6 |
| NCCN/FACT Lymphoma Cancer Symptom Index – 18 Item Version (NFLymSI-18) | Condition-specific (NHL) | 3 | NCT05100862, NCT02626455, NCT02367040 |
| Patient Health Questionnaire (PHQ-9) | Symptom-specific | 3 | NCT05130099, NCT03154710, NCT01483664 |
| Patient-Reported Outcome Measurement Information System (PROMIS-29) | Generic | 3 | NCT03505762, NCT05257785, NCT05259657 |
| Brief Illness Perception Questionnaire (B-IPQ) | PRE | 2 | NTR5953, 2016-004010-10 |
| Cancer Therapy Satisfaction Questionnaire (CTSQ) | PRE | 2 | NCT01724021, NCT01649856 |
| Distress Thermometer (DT) | Symptom-specific | 2 | ACTRN12617000068369, NCT01870479 |
| EORTC QLQ-Elderly (EORTC QLQ-ELD14) | Condition-specific  (cancer in elderly patients) | 2 | NCT05179733, NCT02128061 |
| EORTC QLQ-NHL-Low grade (EORTC QLQ-NHL-LG20) | Condition-specific  (low grade NHL) | 2 | NTR5953, NCT05371093 |
| Functional Living Index-Emesis Questionnaire (FLIE) | Symptom-specific | 2 | Chang 2018, Song 2017 |
| Impact of Event Scale – Revised (IES-R) | Symptom-specific | 2 | NCT00112931, NCT00075946 |
| International Physical Activity Questionnaire (IPAQ) | Functional status and  physical activity | 2 | ACTRN12620000594921, NCT05556239 |
| Rituximab Administration Satisfaction Questionnaire (RASQ) | PRE | 2 | NCT01724021, NCT01649856 |
| Rotterdam Symptom Checklist | Symptom-specific | 2 | NCT05443165, NCT01216683 |
| Self-Rating Anxiety Scale (SAS) | Symptom-specific | 2 | He 2020, Wang 2023 |
| Self-Rating Depression Scale (SDS) | Symptom-specific | 2 | He 2020, Wang 2023 |
| Spielberger State-Trait Anxiety Scale (STAI) | Symptom-specific | 2 | NCT05298293, NCT01870479 |
| WHO Quality of Life Scale (WHOQOL) | Generic | 2 | Liu 2020, Wang 2023 |
| Work Ability Index (WAI) | PRE | 2 | NTR5953, NCT05130099 |
| Active Australia Survey | Functional status and  physical activity | 1 | ACTRN12620000594921 |
| Behavioral regulation in exercise questionnaire (BREQ-3) | Functional status and  physical activity | 1 | NCT05259657 |
| Cancer Behaviour Inventory (CBI) | PRE | 1 | NCT01483664 |
| Cancer Worry Inventory (CWI) | Symptom-specific | 1 | NCT01483664 |
| Cancer Worry Scale (CWS) | Symptom-specific | 1 | NTR5953 |
| Chalder Fatigue Questionnaire (CFQ) | Symptom-specific | 1 | NCT05130099 |
| Consultation and Relational Empathy (CARE) measure | PRE | 1 | NCT01483664 |
| Norwegian Digital Food-Frequency Questionnaire (DIGIKOST-FFQ) | PRE | 1 | NCT05130099 |
| Edmonton Symptom Assessment Scale | Symptom-specific | 1 | Gao 2018 |
| Employment and Health Services Questionnaire (EHSQ) | PRE | 1 | NCT01483664 |
| EORTC QLQ-Chronic Lymphocytic Leukaemia  (EORTC QLQ-CLL17) | Condition-specific  (chronic lymphocytic leukaemia) | 1 | NCT03899337 |
| EORTC QLQ-Chemotherapy-Induced Peripheral Neuropathy (EORTC QLQ-CIPN20) | Side effect-specific | 1 | NCT01324596 |
| EORTC QLQ-Fatigue (EORTC QLQ-FA12) | Symptom-specific | 1 | ACTRN12623000705684 |
| EORTC QLQ-High Dose Chemotherapy  (EORTC QLQ-HDC29) | Condition-specific (cancer) | 1 | NCT06449625 |
| FACIT-Cost | PRE | 1 | ACTRN12617000068369 |
| FACT-Anaemia (FACT-An) | Condition-specific (cancer) | 1 | NCT02165397 |
| FACT-Bone Marrow Transplant (FACT-BMT) | Condition-specific (cancer) | 1 | NCT03417765 |
| FACT-Central Nervous System (FACT-CNS) | Condition-specific (central nervous system cancer) | 1 | NCT00078949 |
| FACT-Neutropenia (FACT-N) | Condition-specific (cancer) | 1 | NCT02044276 |
| Generalised Anxiety Disorder Scale-7 (GAD-7) | Symptom-specific | 1 | NCT05130099 |
| General Quality of Life Inventory (GQOL-74) | Generic | 1 | Luo 2023 |
| Geriatric Depression Scale | Symptom-specific | 1 | NCT04670029 |
| Godin-Shephard Leisure Time Physical Activity Questionnaire (LTPA-Q) | Functional status and  physical activity | 1 | NCT05259657 |
| Haematological Malignancy PROM (HM-PRO) | Condition-specific  (haematologic malignancies) | 1 | NCT05223413 |
| Health Change Questionnaire (HCQ) | PRE | 1 | NCT01014208 |
| Health Education Impact Questionnaire (heiQ) | PRE | 1 | NTR5953 |
| Herth Hope Index (HHI) | PRE | 1 | Lv 2023 |
| HIV Self-Management Scale | Functional status and  physical activity | 1 | Lv 2023 |
| Illness Coping Strategies Scale (ICS) | PRE | 1 | NCT00112931 |
| Illness Perception Questionnaire-Revised (IPQ-R) | PRE | 1 | Munjal 2023 |
| Information Satisfaction Questionnaire (ISQ) | PRE | 1 | NTR5953 |
| Instrumental Activities of Daily Living (IADL) | PRE | 1 | NCT03492775 |
| ItchyQoL | Symptom-specific | 1 | NCT01728805 |
| Kansas City Cardiomyopathy Questionnaire (KCCQ-12) | Condition-specific (heart failure) | 1 | NCT05223413 |
| Lima Happiness Scale | PRE | 1 | NCT04701554 |
| Memorial Symptom Assessment Scale (MSAS) | Symptom-specific | 1 | ACTRN12620000594921 |
| Patient Assessment of Chronic Illness Care (PACIC-20) | PRE | 1 | ACTRN12620000594921 |
| Patient Preference Questionnaire (PPQ) | PRE | 1 | NCT01724021 |
| Patient Satisfaction with the Consultation (PSC) | PRE | 1 | NCT01483664 |
| Perceived Exercise Competence Scale (PCS) | Functional status and  physical activity | 1 | NCT05130099 |
| Petersburg Sleep Questionnaire (PSQI) | PRE | 1 | IRCT20130616013690N6 |
| PROMIS Short-Form Anxiety 7a | Symptom-specific | 1 | ACTRN12623000705684 |
| PROMIS Short-Form Depression 8b | Symptom-specific | 1 | ACTRN12623000705684 |
| Pruritus Quality of Life Score (PQOL) | Symptom-specific | 1 | NCT02811783 |
| Psychological need satisfaction in exercise (PNSE) | Functional status and  physical activity | 1 | NCT05259657 |
| Quality of Life Cancer Survivor (QOL-CS) | Condition-specific (cancer) | 1 | NCT01483664 |
| Short-Form Survivor Unmet Needs (SF-SUNS) | Functional status and  physical activity | 1 | ACTRN12617000068369 |
| Satisfaction With Life Scale (SWLS) | PRE | 1 | NCT05130099 |
| Self-efficacy for walking scale (SEW) | Functional status and  physical activity | 1 | NCT01719562 |
| Short Nutritional Assessment Questionnaire (SNAQ) | PRE | 1 | NCT05556239 |
| Sleep Disturbance visual analogue scale (VAS) | PRE | 1 | NCT03380026 |
| Strategies Used by People to Promote Health (SUPPH) | PRE | 1 | He 2020 |
| Subjective Vitality Scale (SVS) | Functional status and  physical activity | 1 | NCT05130099 |
| Therapy-Induced Neuropathy Assessment Scale (TINAS) | Side effect-specific | 1 | NCT02257567s |
| Trøndelag Health Study (HUNT) Work Status | PRE | 1 | NCT05130099 |
| Verran and Snyder-Halpern Sleep Scale (VSH) | PRE | 1 | Chuang 2017 |
| WHO Quality of Life-HIV (WHOQOL-HIV) | Condition-specific (human immunodeficiency virus) | 1 | Lv 2023 |

NHL: Non-Hodgkin lymphoma; PRE: Patient-reported experience
